# Delineating the *CTBP1*-Related Phenotypic Spectrum: A Review of HADDTS and Atypical Variants

**DOI:** 10.3390/ijms27157065

**Published:** 2026-08-06

**Authors:** Enes Yağız Akdaş, Dingyu Lu, Linshen Zhang, Mingzhen Cheng, Ali Bashiri Dezfouli, Barbara Wollenberg

**Affiliations:** Department of Otolaryngology, Head and Neck Surgery, School of Medicine, Technical University of Munich (TUM), 81675 Munich, Germany

**Keywords:** *CTBP1*, HADDTS, hypotonia ataxia developmental delay tooth enamel defect syndrome, cerebellar atrophy, mitochondrial dysfunction, PXDLS

## Abstract

Hypotonia, Ataxia, Developmental Delay, and Tooth Enamel Defect Syndrome (HADDTS; OMIM #617915) is an ultra-rare autosomal dominant disorder caused by predominantly de novo pathogenic variants in *CTBP1*, encoding a NAD(H)-dependent transcriptional corepressor. We reviewed all HADDTS cases reported from database inception to July 2026, searching PubMed/MEDLINE, Google Scholar, ClinVar, DECIPHER, OMIM, preprint servers, and the HADDTS Foundation, identifying 25 peer-reviewed cases from at least 11 countries; registries indicate at least 50 known individuals. Global developmental delay and language impairment were universal (25/25, 100%), followed by intellectual disability (24/25, 96%), hypotonia (22/25, 88%), ataxia and enamel defects (19/25, 76% each), cerebellar atrophy (18/25, 72%), feeding difficulties (15/25, 60%), myopathy (15/25, 60%), regression (10/25, 40%), oculomotor apraxia (7/25, 28%), scoliosis (6/25, 24%), respiratory chain dysfunction (5/25, 20%), skeletal anomalies (4/25, 16%), and seizures (2/25, 8%). The recurrent p.Arg342Trp (NM_001328.2; p.Arg331Trp, MANE Select NM_001012614.2) accounts for 84%, with severity from mild impairment to profound disability. In all four non-recurrent-variant carriers the canonical tetrad was incomplete; seizures and classifying skeletal anomalies occurred only in that group. Mutant CTBP1 acts dominant-negatively and heterodimerises with the essential paralog CTBP2, explaining the multisystem severity. HADDTS is a neurodevelopmental-mitochondrial overlap disorder; registries, mitochondrial evaluation, and allele-specific therapies are priorities.

## 1. Introduction

Hypotonia, Ataxia, Developmental Delay, and Tooth Enamel Defect Syndrome (HADDTS; OMIM #617915) is an extremely rare autosomal dominant developmental disorder caused by predominantly de novo pathogenic variants in *CTBP1* (MIM 602618) on chromosome 4p16 [1,2]. The *CTBP1* gene encodes C-terminal Binding Protein 1 (CTBP1), a highly conserved NAD(H)-dependent transcriptional corepressor that links cellular metabolic state to chromatin remodelling by recruiting histone-modifying enzymes, including deacetylases and methyltransferases, to nucleate corepressor complexes that drive epigenetic gene silencing at target promoters [1]. The CTBP1 protein is implicated in neurodevelopment, neuronal differentiation, apoptosis, cell-fate determination, stem-cell renewal, muscle biogenesis, and mitochondrial function, integrating the NAD^+^/NADH ratio to coordinate transcriptional programmes relevant to both development and oncogenesis [1,3,4,5,6,7,8,9]. Beyond its nuclear role, cytoplasmic CTBP1 contributes to Golgi membrane dynamics, vesicular trafficking, and, in neurons, synaptic function [7,10].

HADDTS was first described in 2016 by Beck et al., who identified a recurrent de novo missense variant (c.991C>T; p.Arg331Trp on the short isoform NM_001012614.2, the current MANE Select transcript, equivalent to c.1024C>T; p.Arg342Trp on the long isoform NM_001328.2) in four unrelated children sharing a strikingly similar neurodevelopmental phenotype [2]. Following this landmark report, additional patients have been described from the USA, UK, India, Iran, Australia, Belgium, China, Japan, Turkey, Bulgaria, and Poland [2,9,11,12,13,14,15,16,17,18,19,20,21,22]. As of July 2026, 25 patients with confirmed or likely pathogenic *CTBP1* variants have been reported in the peer-reviewed literature across at least 11 countries and 14 publications. The majority carry the recurrent p.Arg342Trp variant (NM_001328.2; p.Arg331Trp on NM_001012614.2), alongside an expanding spectrum of atypical, non-recurrent variants. The HADDTS Foundation, which also serves as a patient-advocacy group, indicates that additional genetically confirmed individuals remain unpublished [23].

A critical molecular finding with implications for severity is the demonstration that mutant CTBP1 retains the ability to homo- and heterodimerize with wild-type CTBP proteins [17]. This raises the possibility that the dominant-negative mutant not only disrupts CTBP1-mediated repression but also interferes with CTBP2-dependent gene regulation. Because homozygous loss of *Ctbp2* is embryonic lethal in mice [24], such trans-paralog interference could substantially amplify the consequences of the mutation, providing a molecular explanation for why HADDTS produces a phenotype far more severe than *CTBP1* loss alone would predict. Notably, predicted loss-of-function *CTBP1* variants, including homozygotes, are tolerated without clinical consequence in population databases such as gnomAD, consistent with this model [25] (Supplementary Table S1). This review synthesises all available published cases from English and non-English sources to provide an exhaustive clinical reference, with tabulated patient data, a mutation registry, phenotypic frequency analysis, and an overview of molecular pathomechanisms and emerging therapies.

## 2. Methods

This review was conducted and reported in accordance with the PRISMA 2020 guidelines [26] where applicable (Supplementary Document S1). Because the available evidence consisted of single-patient case reports, small case series, and genotype-phenotype descriptions, no meta-analysis was performed; the work is therefore reported as a comprehensive review of all published cases rather than as a systematic review with meta-analysis, although the PRISMA 2020 flow diagram and the a priori eligibility criteria have been retained as transparency aids. Findings were synthesised descriptively, with every frequency expressed over the full set of 25 published patients, so that all denominators are identical and all frequencies are minimum estimates. Two reviewers independently searched PubMed/MEDLINE, Google Scholar, ClinVar, OMIM, DECIPHER, and the Simons Searchlight registry from database inception to July 2026, supplemented by preprint servers (bioRxiv, medRxiv), the HADDTS Foundation, conference abstracts, and hand-searching of reference lists. Search terms combined controlled-vocabulary and free-text terms, with “CTBP1” always paired (AND) with at least one of the following (OR): “HADDTS”, “hypotonia ataxia developmental delay tooth enamel defect syndrome”, “Arg342Trp”, “Arg331Trp”, “R331W”, or “R342W”. Population allele-frequency data were extracted from gnomAD v4.1.1.

**Variant nomenclature**: All variants are described according to current HGVS recommendations, and the reference transcript is stated explicitly with every variant designation. Because the *CTBP1* literature reports variants against two different reference sequences, the MANE Select transcript NM_001012614.2 (CTBP1-S, 429 residues) is used here as the primary reference, with the corresponding designation on the long isoform NM_001328.2 (CTBP1-L, 440 residues) given alongside it at every mention in the running text and in Table 1; Table 2 lists the designations used in the source publications, with their MANE Select equivalents given in Table 1. Residue numbering on NM_001328.2 exceeds that on NM_001012614.2 by 11 amino acids (33 nucleotides) throughout the shared reading frame.

**Variant database interrogation:** ClinVar [27] was queried with the gene-level term *CTBP1* and by direct interrogation of the aggregate record for the recurrent variant (Variation ID 225758; rs869320802). For every submission we recorded the submitting laboratory, the germline classification, the review status, the date of last evaluation, the condition asserted, and the evidence cited, in order to identify genetically confirmed individuals not represented in the peer-reviewed literature; submissions were cross-checked against the included publications to avoid double-counting (Supplementary Table S2). Submissions classified as of uncertain significance, likely benign, or benign, and somatic submissions, were recorded but not counted as cases. In addition, DECIPHER [28] was searched for *CTBP1* sequence variants (Supplementary Table S2) and for copy-number variants overlapping the *CTBP1* locus (Supplementary Table S3). For every record retrieved we noted the DECIPHER patient identifier, the variant and its predicted consequence, the inheritance status, the contributing centre, and the pathogenicity assigned by that centre.

**Eligibility criteria:** Eligibility criteria were defined a priori. Reports were included if they (i) were peer-reviewed primary studies (case reports, case series, or cohorts) describing one or more individuals with a confirmed pathogenic or likely pathogenic *CTBP1* variant, and (ii) provided extractable individual-level clinical, genetic, neuroimaging, or pathological data; sources in any language were eligible, with non-English articles translated and independently verified. Reports were excluded if they (i) were functional, molecular, or animal studies without primary human patient data; (ii) were reviews, editorials, or other secondary sources containing no original cases; (iii) duplicated individuals already reported elsewhere; or (iv) were non-peer-reviewed records from which no patient-level data could be extracted. Where the same individual appeared in more than one publication, data were consolidated to avoid double-counting.

**Figure 1:**
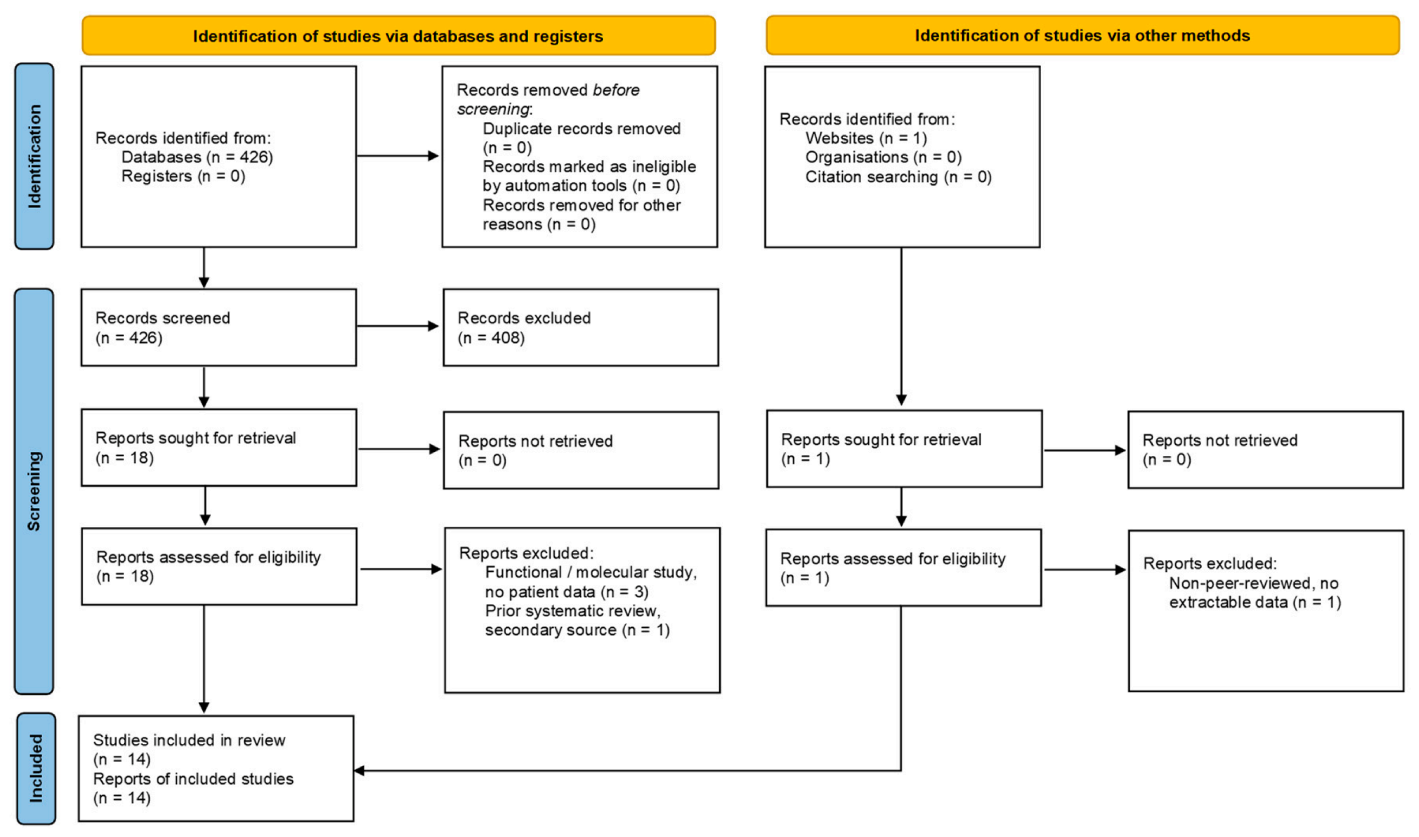
PRISMA 2020 flow diagram of study identification, screening, and inclusion. Of 426 records identified through database searching and 1 through other sources, 14 studies (25 patients) met the eligibility criteria and were included in the qualitative synthesis. Adapted from Page et al. (BMJ 2021;372:n71) under CC BY 4.0 [26].

**Study selection:** Two reviewers (E.Y.A. and D.L.) independently screened titles and abstracts and then assessed the full texts of potentially eligible reports against these criteria, resolving disagreements by consensus. The selection process is summarised in the PRISMA 2020 flow diagram (Figure 1). Database searches returned 426 records, none of which were removed as duplicates or by automation before screening; 408 were excluded at title and abstract screening because they addressed *CTBP1* in oncological, cell-biological, or other contexts unrelated to HADDTS; were basic functional, structural, or animal studies without human patient-level data; were reviews, editorials, commentaries or conference material containing no original cases; concerned other genes or unrelated disorders retrieved by the free-text search terms; described individuals already reported in another retrieved record; or contained no extractable individual-level data. Eighteen reports were retrieved and assessed for eligibility, of which four were excluded (3 functional or molecular studies without patient data and one prior systematic review). One further report identified through other sources was assessed and excluded as a non-peer-reviewed record without extractable data. Fourteen studies, reporting 25 individual patients, met all criteria and were included in the qualitative synthesis.

## 3. Results and Discussion

### 3.1. Genetics of CTBP1 and HADDTS

#### 3.1.1. Gene Structure and Chromosomal Location

*CTBP1* maps to chromosome 4p16.3 (GRCh37: chr4:1,205,233-1,242,918) and encodes two well-characterised isoforms that are identical except at their extreme N-termini: a long isoform of 440 residues (CTBP1-L; NM_001328.2) and a short isoform of 429 residues (CTBP1-S; NM_001012614.2, the current MANE Select transcript), differing by 13 N-terminal residues but only 11 in overall length [1]. The CTBP1 protein shares ~80% amino acid identity with its paralog CTBP2 (chromosome 10q26); both are expressed across human tissues but differ in developmental timing, nuclear localisation architecture, and subcellular compartmentalisation, and are capable of forming heterodimers [1,24,29]. Critically, while *Ctbp1*-knockout mice are viable, *Ctbp2* knockout is embryonic lethal at E10.5 due to aberrant extraembryonic development, demonstrating essential, non-redundant CTBP2 functions and underscoring the potential severity of any interference with CTBP2 [24].

**Figure 2:**
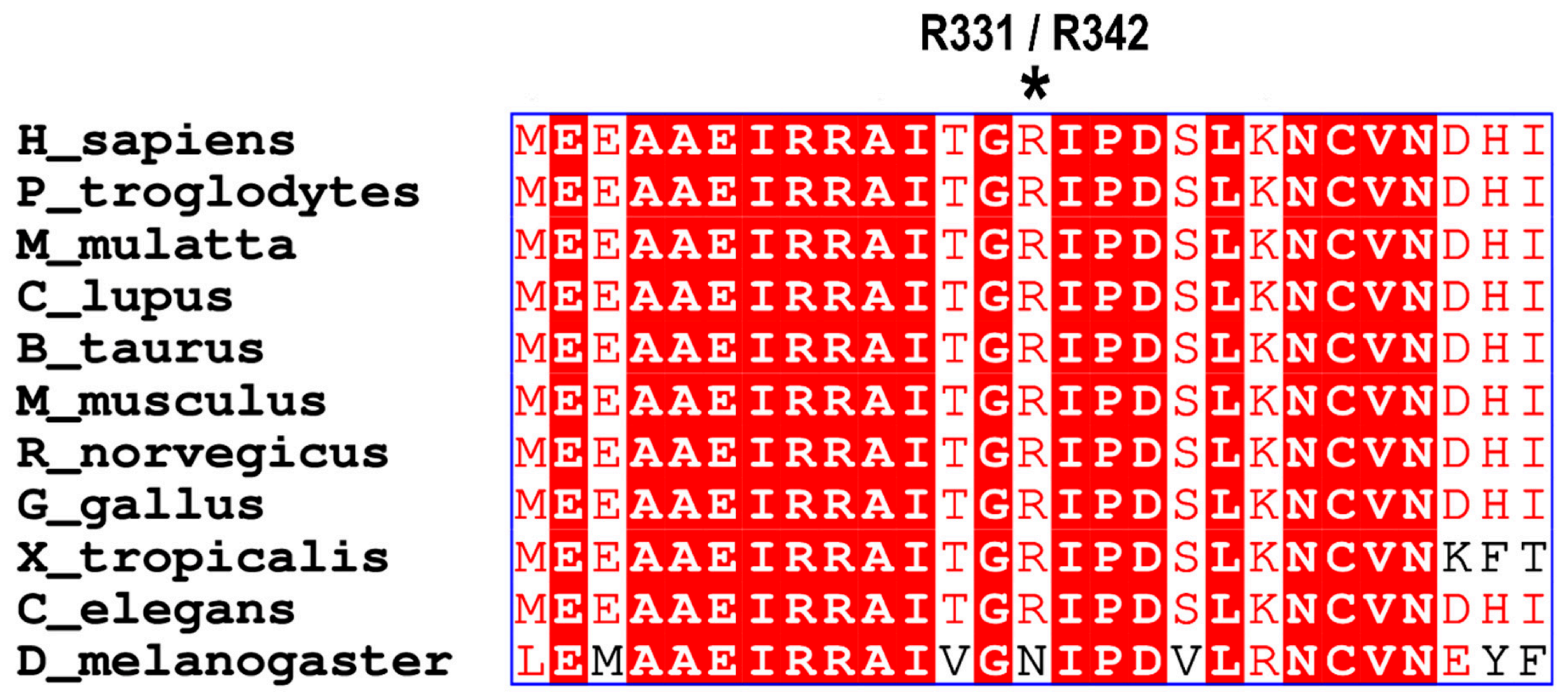
Evolutionary conservation of the PXDLS binding cleft region across eukaryotic CTBP1 orthologs. The R331/R342 residue, site of the recurrent HADDTS-causing variant p.Arg331Trp (NM_001012614.2, MANE Select; p.Arg342Trp on NM_001328.2, showed with *), is invariant from C. elegans to H. sapiens, underscoring its structural and functional importance in the PXDLS protein–protein interaction interface. Generated using ESPript v3.2; red background with white text indicates residues identical across all aligned sequences, and white background with red or black text indicates non-conserved positions (black denotes the least conserved residue at that position). Nucleotide-level constraint at the corresponding genomic position (chr4:1,213,028, GRCh38; chr4:1,206,816, GRCh37) is at or near the ceiling of the phyloP and phastCons scales: phyloP100way = 7.20 (track maximum 7.53), phastCons100way = 1.00; GERP++ RS is 2.8, an intermediate value on that scale. Metrics were retrieved from dbNSFP via the UCSC Genome Browser (phyloP and phastCons from the hg38 100-way vertebrate alignment; GERP++ RS from the hg19 mammalian alignment).

At the structural level, CTBP1 is organised into three functionally distinct domains [1,30]: (**i**) the N-terminal NAD(H)-binding substrate domain, conferring homology to the D-isomer-specific 2-hydroxyacid dehydrogenase (D2-HDH) superfamily and acting as a metabolic sensor that links redox state to repressor activity; (**ii**) the central Arg-Arg-Thr (RRT) domain, mediating NAD(H)-dependent homo- and heterodimerisation, including with CTBP2, essential for full corepressor activity; and (**iii**) the evolutionarily conserved Pro-X-Asp-Leu-Ser (PXDLS) binding cleft, the principal protein–protein interaction interface through which CTBP1 recruits chromatin-modifying cofactors to nucleate corepressor complexes. All HADDTS-associated pathogenic variants identified to date reside within or adjacent to the PXDLS cleft or the NAD(H)-binding domain, underscoring their indispensable regulatory role [2,9,11,12,13,14,15,16,17,18,19,20,21,22]. The PXDLS cleft is strikingly conserved, with Arg342 (NM_001328.2), equivalent to Arg331 (NM_001012614.2), invariant from *C. elegans* to humans (Figure 2).

#### 3.1.2. Pathogenic Variants and Mutational Hotspot

Five distinct pathogenic or likely pathogenic *CTBP1* variants have been reported (Table 1).

**(1) c.991C>T; p.Arg331Trp (NM_001012614.2, MANE Select), equivalent to c.1024C>T; p.Arg342Trp (NM_001328.2)** is the recurrent missense variant in exon 9, affecting the α5-helix that forms the C-terminal wall of the PXDLS-binding cleft. It has been reported in 21 of 25 published patients (84%) and is classified as pathogenic (ClinVar Variation ID 225758, aggregate classification pathogenic; dbSNP rs869320802). The phenotype is classic HADDTS across its full documented severity range. The affected CpG dinucleotide is prone to spontaneous deamination of methylated cytosine, explaining its recurrence across unrelated individuals of diverse ethnicities [2,9,11,12,14,15,16,17,18,20,22].

**(2) c.1282_1283delCA; p.Gln428ValfsTer84 (NM_001012614.2, MANE Select), reported as c.1315_1316delCA; p.Gln439ValfsTer84 (NM_001328.2)** is a two-nucleotide deletion in exon 10 causing a frameshift and a premature termination codon within the C-terminal PXDLS domain. It has been reported in one of 25 published patients (4%), an Iranian male, and is classified as pathogenic; the reporting authors did not specify the ACMG criteria applied. The phenotype reproduces most classic HADDTS features but lacks hypotonia and includes a single myoclonic seizure [13].

**(3) c.338C>T; p.Ser113Phe (NM_001012614.2, MANE Select), reported as c.371C>T; p.Ser124Phe (NM_001328.2)**, is a missense variant in exon 5, within the NAD(H)-binding (substrate-binding) domain. It has been reported in one of 25 published patients (4%), a Chinese female, and is classified as likely pathogenic by ACMG criteria (PS2, PM2-supporting, PP2, PP3). The phenotype is atypical and non-canonical, with developmental delay, dysmorphism, and pectus excavatum but neither ataxia nor enamel defects [21].

**(4) c.74G>C; p.Arg25Pro (NM_001012614.2, MANE Select), reported as c.107G>C; p.Arg36Pro (NM_001328.2)** is a missense variant in exon 3, within the PXDLS-binding cleft. It has been reported in one of 25 published patients (4%), a 20-year-old Japanese male, and is classified as pathogenic; the reporting authors did not specify the ACMG criteria applied. The phenotype is atypical, comprising severe intellectual disability, atrial septal defect, and dysmorphism; the authors explicitly noted that it did not fulfil HADDTS criteria [11].

**(5) c.970T>G; p.Ser324Ala (NM_001012614.2, MANE Select), equivalent to c.1003T>G; p.Ser335Ala (NM_001328.2)** is a missense variant in exon 8, within the substrate-binding subdomain of the D2-HDH fold, seven residues upstream of the Arg342/Arg331 hotspot and immediately adjacent to the α5-helix framing the PXDLS cleft. It has been reported in one of 25 published patients (4%), a 1-year-old Turkish male of consanguineous parents; it was confirmed de novo by parental testing and is classified as likely pathogenic (PS2, PM2, PP2). The patient presented a West syndrome-like phenotype that broadens the recognised spectrum [19].

#### 3.1.3. CTBP1 p.Arg342Trp (NM_001328.2) Mechanism

**Figure 3:**
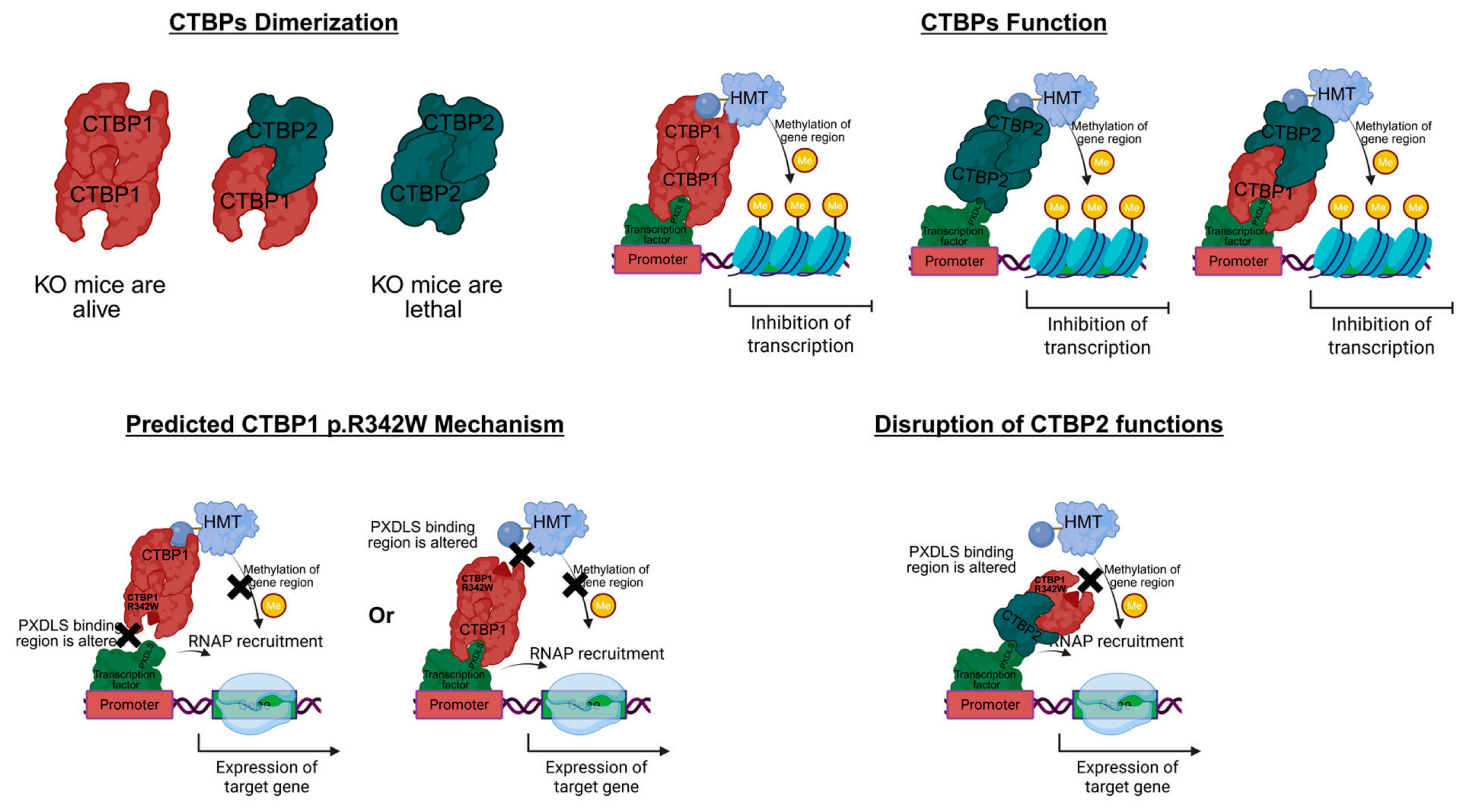
Proposed model of CTBP1/CTBP2 dimerisation and functional disruption by dominant-negative CTBP1 p.R342W. CTBP1 forms CTBP1–CTBP1 homodimers and CTBP1–CTBP2 heterodimers, whereas CTBP2 forms homodimers; in vivo, loss of CTBP1 does not affect survival, whereas loss of CTBP2 is embryonic lethal. CTBP proteins repress transcription by binding PXDLS-containing cofactors and recruiting chromatin-modifying complexes to target promoters. p.R342W is predicted to impair repression by (i) altering the PXDLS-binding region and reducing cofactor recruitment, and (ii) forming dysfunctional heterodimers that interfere with CTBP2-dependent repression despite preserved CTBP2 expression, causing inappropriate activation of CTBP2 target genes. Created in BioRender. Hachani, K. (2026), https://BioRender.com/yw9f1pc.

The pathogenic mechanism of p.Arg342Trp (NM_001328.2; p.Arg331Trp on NM_001012614.2) is not simple haploinsufficiency, as healthy individuals carry predicted loss-of-function *CTBP1* variants without developmental phenotypes [25]. Instead, the variant acts through a dominant-negative mechanism, producing a mutant corepressor that binds wild-type CTBP1 but exhibits aberrant cofactor recruitment, deranging repression at developmentally critical loci (Figure 3) [17]. Using flag-immunoprecipitation mass spectrometry of cells expressing wild-type versus mutant CTBP1, Beck et al. (2019) demonstrated significantly reduced co-immunoprecipitation of multiple chromatin-modifying factors from the mutant allele, indicating impaired assembly of the corepressor complex [17]. Transcriptome analysis of patient-derived cells revealed upregulation of apoptotic, immune, and metabolic gene networks consistent with loss of normal repression, and patient fibroblasts showed enhanced apoptosis sensitivity under glucose deprivation with upregulation of the pro-apoptotic factor *NOXA*, linking the metabolic-sensing function of CTBP1 to a cell-survival phenotype [17,31].

Crucially, mutant CTBP1 retains the capacity to heterodimerise with CTBP2 [17]. Because CTBP1 and CTBP2 normally heterodimerise through the RRT domain to coordinate repression at shared loci [24,30], incorporation of a mutant subunit could generate dysfunctional complexes with impaired cofactor recruitment, extending the consequences beyond CTBP1-homodimer targets to CTBP1–CTBP2 heterodimer targets. Given that *Ctbp2* loss is embryonic lethal [24], partial CTBP2 compromise through this trans-paralog interference could substantially amplify phenotypic severity, including the neurodevelopmental, mitochondrial, and muscular features that are disproportionate to haploinsufficiency alone. This model explains why predicted loss-of-function *CTBP1* variants are population-tolerated while the dominant-negative p.Arg342Trp (NM_001328.2) produces devastating multisystem disease [17,25].

#### 3.1.4. Inheritance and Recurrence Risk

HADDTS follows autosomal dominant inheritance, with the overwhelming majority of cases arising de novo ADDIN EN.CITE [2,9,11,12,13,14,15,16,17,18,19,20,21,22]. One exceptional case (Beck 2016, Patient 1) demonstrated maternal germline mosaicism at an estimated ~5.3% allele fraction, rendering the mother phenotypically unaffected but conferring a non-negligible recurrence risk for future pregnancies, an important consideration for genetic counselling, as apparently de novo variants may rarely reflect parental gonadal mosaicism [2]. The recurrence risk for offspring of an affected individual would theoretically be 50%, though reproduction has not been reported given the severity of the phenotype. The p.Arg342Trp (NM_001328.2) mutation is absent from gnomAD v4.1.1 and all major population databases, consistent with strong negative selection against the dominant-negative allele [25]. All peer-reviewed cases are presented in Table 2.

### 3.2. Clinical Phenotype and Natural History

Since its delineation in 2016 [2], the clinical and pathological spectrum of HADDTS has expanded considerably through additional case reports, functional studies, and cohort reanalysis [2,3,9,11,12,13,14,15,16,17,18,19,20,21,22,31]. Although defined by four core features, hypotonia, ataxia, developmental delay, and tooth enamel defects, HADDTS is a multisystem disorder with substantial inter-individual variability, even among patients harbouring identical variants. Among 23 patients with documented sex, 15 (65%) are males and eight (35%) females; this imbalance likely reflects ascertainment bias rather than a true predisposition, as the disorder is autosomal dominant [2,11,17].

#### 3.2.1. Core Clinical Features

**Hypotonia** is the most consistent and frequently the earliest presenting feature, typically noted in the neonatal period or early infancy and affecting both axial and appendicular musculature [2,9,11,12,13,14,15,16,17,18,19,20,21,22]. In the original Beck et al. (2016) cohort all four patients showed clinically significant hypotonia, with severity from mild to profound; Sommerville (2017), Ozaki (2020), Kadhim (2023), and Marco (2025) similarly described severe generalised hypotonia from early infancy contributing to motor delay [2,9,12,15,18,22]. The axial component is often most functionally limiting, compromising head control, unsupported sitting, and trunk stability; in severe cases the combination of axial hypotonia and appendicular weakness prevents independent sitting or standing, whereas in milder cases tone improves sufficiently to permit a broad-based, ataxic gait [2,9,14,18,22]. Peripheral hypotonia manifests as decreased limb resistance, joint hypermobility, and diminished deep-tendon reflexes [2,18]. Pathophysiology is multifactorial: disruption of CTBP1-dependent programmes impairs neuronal, cerebellar, and skeletal-muscle development, while superimposed mitochondrial respiratory chain defects and congenital fibre-type disproportion add a peripheral myopathic component in at least a subset [3,4,9,12,18,22,31].

**Global developmental delay** is a universal feature, encompassing motor, cognitive, and verbal domains [2,9,11,12,13,14,15,16,17,18,19,20,21,22]. Delays in gross-motor milestones, head control, rolling, unsupported sitting, pulling to stand, independent ambulation, are typically the earliest recognised concern and often prompt initial evaluation. Intellectual disability accompanies motor delay in the vast majority, ranging from moderate to severe, and speech is severely impaired, with several patients having no functional expressive language and communicating through gestures, vocalisations, or augmentative and alternative communication [2,9,14,15,18,22]. A clinically important exception is Beck 2016 Patient 3 (female, 9 y), who achieved normal cognition, age-appropriate speech, and functional literacy despite the classic p.Arg342Trp (NM_001328.2) variant, the mildest known phenotype and direct evidence that the same variant can produce markedly different neurocognitive outcomes, presumably reflecting genetic-background modifiers, developmental variation, or epigenetic factors [2,17]. A concerning subset shows developmental regression, loss of previously acquired motor, language, or cognitive skills, exemplified by the Ozaki et al. (2020) 14-year-old who experienced severe psychomotor regression with progressive cerebellar atrophy, and by the Sommerville (2017) patient who lost language skills by ~age 4 [2,9,12,17]. Proposed contributors include progressive mitochondrial dysfunction, ongoing disruption of activity-dependent gene regulation, and cumulative cellular stress [9,12,17,31].

**Cerebellar ataxia** is a core feature and the principal determinant of motor function and ambulatory status [2,9,11,12,13,14,15,16,17,18,19,20,21,22]. In ambulatory patients, gait is characteristically broad-based, unsteady, and poorly coordinated; limb ataxia may manifest as dysmetria, dysdiadochokinesia, and intention tremor [2,13,14,17]. Non-ambulation (wheelchair dependence) was documented in two Beck 2016 patients and in several subsequent cases (Sommerville 2017, Ozaki 2020), where severe axial hypotonia, ataxia, and superimposed myopathy combine, carrying secondary consequences including progressive scoliosis, joint contractures, osteoporosis, and respiratory complications [2,9,12,18]. Oculomotor apraxia, impaired voluntary saccades with compensatory head thrusting, was documented in five of the 12 patients in the Beck (2019) cohort and in subsequent reports; its presence reinforces cerebellar localisation and prompts consideration of ataxia-oculomotor apraxia types 1 and 2, ataxia-telangiectasia, and Joubert syndrome [17,18].

**Tooth enamel defects**, present in 19 of 25 patients (76%), provide an important diagnostic clue, manifesting as hypoplastic, soft, fragile, or discoloured enamel of both primary and permanent dentitions, susceptible to accelerated wear, chipping, and carious destruction, with root resorption in some patients [2,13,14,15,17,18]. The molecular basis presumably reflects CTBP1’s role in regulating ameloblast differentiation and amelogenesis-related gene expression [2,17]. Notably, two patients carrying the identical p.Arg342Trp (NM_001328.2) variant, Sommerville (2017) and Ozaki (2020), had no clinically detectable enamel defects, representing significant outliers that underscore phenotypic variability even for core features; it remains unknown whether subclinical abnormalities were present or whether the enamel phenotype is subject to incomplete penetrance. Absent enamel involvement should therefore not exclude the diagnosis in a patient otherwise fulfilling neurodevelopmental criteria [9,12,18].

#### 3.2.2. Neuroimaging

Brain MRI is an essential component of the diagnostic evaluation and reveals a consistent pattern of cerebellar involvement [2,9,11,12,13,14,15,16,17,18,19,20,21,22]. Cerebellar atrophy, ranging from mild volume loss of the vermis and hemispheres to severe, progressive hypoplasia, is the most reproducible finding and correlates closely with ataxia severity. The Japanese patient of Ozaki et al. (2020) demonstrated progressive cerebellar atrophy on serial imaging, with worsening volume loss between childhood and age 14, providing direct evidence for an ongoing neurodegenerative process in a subset rather than a purely static malformation [12]. Supratentorial structures are generally better preserved, though thin corpus callosum, mild cortical volume loss, and nonspecific white matter changes occur in isolated cases [2,16,17]. The cerebellar predominance has been illuminated transcriptomically: Lee and Ezekiel (2025), using the Allen Brain Cell Atlas, confirmed that *CTBP1* and *CTBP2* are highly expressed in the upper rhombic lip (URL) supercluster, the embryonic germinative zone giving rise to cerebellar granule-cell precursors, the most abundant cerebellar neurons [32]. Lee et al. (2025), using isogenic iPSC-derived neurons carrying p.R342W, demonstrated downregulation of transcription factors governing URL and granule-cell development, including *OLIG3* and *BARHL1*, in both heterozygous and homozygous mutant cells, validating this anatomical prediction at the molecular level [3,32].

#### 3.2.3. Mitochondrial Dysfunction

Mitochondrial respiratory chain dysfunction is a recurrent and probably underdiagnosed feature [9,16,18,20]. It was first documented by Sommerville et al. (2017), who found markedly decreased complex I and IV activities in skeletal muscle with relative preservation of complexes II and III, a pattern characteristic of a nuclear gene defect affecting mitochondrial biogenesis rather than a primary mtDNA mutation [9,16]. Wong et al. (2022) reported concordant biochemical abnormalities with clinical improvement on a mitochondrial cofactor cocktail, and Kadhim et al. (2023) provided detailed histochemical and ultrastructural evidence (COX heterogeneity, vacuolated mitochondria with disorganised cristae, subsarcolemmal aggregates) [16,18]. Mechanistically, the mitochondrial proteome is overwhelmingly nuclear-encoded (~1100 of ~1300 proteins), and dominant-negative disruption of CTBP1, compounded by dysfunctional CTBP1–CTBP2 heterodimers affecting metabolic gene networks, is hypothesised to dysregulate their transcription [9,17,24,29]. Targeted evaluation, including serum and CSF lactate, circulating biomarkers (e.g., FGF-21, GDF-15), and, in selected cases, muscle biopsy with respiratory chain enzymology, may be considered in patients with features suggestive of mitochondrial dysfunction [9,16,18].

Most recently, Ivanov et al. (2026) provided the first real-time, non-invasive characterisation by applying Seahorse XFp respirometry to peripheral blood mononuclear cells (PBMCs) from a 10-year-old Bulgarian female carrying p.Arg342Trp (NM_001328.2) [20]. Compared with controls, the patient’s PBMCs showed markedly reduced maximal respiration (28.77 vs. 52.36 pmol/min), profoundly impaired spare respiratory capacity (107% vs. 261% of basal), and reduced extracellular acidification rate (ECAR), indicating a global bioenergetic and glycolytic deficit concordant with the muscle biopsy findings of earlier reports [9,16,18,20]. After one year of cofactor supplementation (coenzyme Q10, riboflavin, vitamin C, vitamin E), maximal respiration and spare capacity recovered toward control values and respiratory infections decreased, yet no neurological or developmental improvement occurred [20]. This dissociation between biochemical reversibility and clinical refractoriness indicates that mitochondrial dysfunction is one, but not the principal, axis of HADDTS pathogenesis, and that the ECAR reduction places the metabolic phenotype beyond OXPHOS, consistent with CTBP1’s role as a NAD(H)-sensing global metabolic regulator [20]. Respirometry in PBMCs therefore offers a minimally invasive, longitudinally repeatable readout for cofactor-response monitoring.

#### 3.2.4. Muscular Pathology

Recognition that HADDTS is associated with primary structural myopathy represents a significant expansion of its pathological spectrum, documented across three independent publications [12,16,18]. Ozaki et al. (2020) reported the first case of congenital fibre-type disproportion (CFTD) in HADDTS: muscle biopsy at age 4 in a Japanese male carrying the recurrent variant revealed marked disproportion between type 1 and type 2 fibres, leading to an initial CFTD diagnosis before the identification of the *CTBP1* variant. As CFTD is traditionally classified among congenital myopathies, this finding suggests it may represent an early histopathological manifestation within the spectrum of CTBP1-related muscle disease and underscores the importance of considering *CTBP1* in unexplained congenital myopathy combined with neurodevelopmental features [12]. Kadhim et al. (2023) provided the most comprehensive histomyopathological characterisation, in a Belgian male: light microscopy revealed endomysial fibrosis, marked fibre-size variability, active degeneration and necrosis with macrophage infiltration, internal nuclei, and vacuolar myopathy with rimmed vacuoles; Oil-Red-O staining showed lipid accumulation suggesting impaired fatty-acid oxidation; fibre typing demonstrated pronounced type 1 predominance; and electron microscopy showed vacuolated mitochondria, disorganised cristae, myofibrillar disruption, and autophagic debris, supporting a dystrophic-mitochondrial phenotype [18]. Wong et al. (2022) similarly reported dystrophic and vacuolar changes with mitochondrial ultrastructural abnormalities [16]. Beyond intrinsic mitochondrial dysfunction, CTBP1 links neuronal activity to muscle transcriptional and metabolic programmes, and its loss promotes denervation-like states with metabolic reprogramming, altered respiratory chain gene expression, and mitochondrial network remodelling; impaired activity-dependent maintenance of muscle homeostasis may therefore contribute to mitochondrial abnormalities and degeneration independently of primary mitochondrial defects [7]. Collectively, muscle weakness in HADDTS is not solely attributable to central dysfunction but involves a substantial peripheral myopathic component, likely driven by combined mitochondrial dysfunction and disrupted neuromuscular activity-dependent regulation, providing a rationale for therapies targeting both mitochondrial function and muscle integrity [12,16,18].

#### 3.2.5. Prognosis and Disease Course

The natural history of HADDTS remains incompletely characterised owing to its extreme rarity, but morbidity is substantial across the spectrum. Even in the mildest documented case (Beck 2016, Patient 3), ataxic gait and motor impairment impose functional limitations and the enamel defect requires ongoing dental management; in the majority of patients morbidity is far greater, encompassing non-ambulation, severe intellectual disability, absent functional speech, dysphagia necessitating gastrostomy, recurrent respiratory infections, progressive scoliosis, and, in a subset, seizures [2,9,11,12,13,14,15,16,17,18,19,20,21,22]. In severe cases HADDTS may be associated with reduced life expectancy, although systematic outcome data are lacking; based on the documented clinical features, potential complications include aspiration-related events from dysphagia, respiratory compromise from neuromuscular weakness or skeletal deformity, and sequelae of profound neurological impairment. The documentation of progressive mitochondrial dysfunction and developmental regression, notably in the Ozaki (2020) and Sommerville (2017) cases, raises the possibility of a neurodegenerative trajectory in a subset, while the determinants of severity (allelic effects, genetic modifiers, epigenetic variation, or stochastic developmental events) remain unknown [2,9,12,17]. Patient-level features are presented in Table 3 and aggregate frequency and severity in Table 4. Prospective natural history studies are a high priority and should incorporate serial neurological, cognitive, and motor assessment; longitudinal neuroimaging with quantitative volumetry; serial biomarker monitoring of mitochondrial function; and systematic assessment of nutritional status, respiratory function, and quality of life, with the HADDTS Foundation and Simons Searchlight registries providing critical infrastructure [23,33].

#### 3.2.6. Aggregate Phenotypic Frequencies and Genotype–Phenotype Correlations

Across the 25 published patients, global developmental delay and language impairment were universal (25/25, 100%), followed by intellectual disability (24/25, 96%), hypotonia (22/25, 88%), cerebellar ataxia and tooth enamel defects (19/25, 76% each), cerebellar atrophy on MRI (18/25, 72%), feeding difficulties or failure to thrive (15/25, 60%), muscle weakness or documented myopathy (15/25, 60%), developmental regression (10/25, 40%), oculomotor apraxia (7/25, 28%), scoliosis (6/25, 24%), respiratory chain dysfunction (5/25, 20%), skeletal anomalies including pectus excavatum (4/25, 16%), and seizures (2/25, 8%). Because reporting in the source literature is incomplete, features that were not mentioned were counted as not documented rather than as absent, so these figures are minimum estimates; mitochondrial dysfunction, oculomotor apraxia, and enamel defects in children who have not yet erupted the permanent dentition are the most likely to be under-ascertained.

Three features distinguish the disorder from wider differential of infantile hypotonia with cerebellar signs. Hypoplastic or dystrophic enamel affecting both dentitions, together with ataxia and global developmental delay, is near-specific for *CTBP1*-related disease. Oculomotor apraxia with compensatory head thrusting (7/25, 28%) narrows the differential towards ataxia-oculomotor apraxia types 1 and 2, ataxia–telangiectasia, and Joubert syndrome, each excludable on other grounds. A primary myopathic component (15/25, 60%), including congenital fibre-type disproportion, is unusual alongside a cerebellar and neurodevelopmental phenotype and should prompt consideration of *CTBP1* when a congenital myopathy is identified in a child with global developmental delay.

Genotype–phenotype correlation is constrained by the dominance of a single allele: 21 of 25 patients (84%) carry the recurrent p.Arg342Trp/p.Arg331Trp (NM_001328.2/NM_001012614.2) variant and the remaining four each carry a private variant. Within the recurrent-variant group hypotonia and global developmental delay were documented in all 21 (100%), with ataxia and enamel defects in the large majority, whereas the canonical tetrad was incompletely expressed in all four patients with non-recurrent variants. Two features separate the groups. Epilepsy was documented in only two patients overall (8%), both outside the recurrent-variant group (the p.Gln439ValfsTer84 (NM_001328.2) carrier with a single myoclonic seizure and the p.Ser335Ala (NM_001328.2) carrier with West syndrome), and Beck et al. (2019) recorded an explicit absence of seizures across their 12 patients. Pectus excavatum and the other classifying skeletal anomalies were likewise confined to the four carriers of non-recurrent variants (4/4, 100%). Conversely, documented mitochondrial dysfunction (5/25, 20%) and biopsy-proven myopathy have been described only in recurrent-variant carriers, although muscle biopsy and respiratory chain enzymology have been performed almost exclusively in that group.

The widest variation lies within rather than between genotypes. Among carriers of the recurrent variant, phenotypes extend from Beck 2016 Patient 3, whose cognition was age-appropriate and whose principal limitation was an ataxic gait, to patients who never sat independently, lost acquired motor and language skills, and required gastrostomy feeding and correction of kyphoscoliosis; intellectual disability, absent in that one patient alone, ranged from mild to profound in the remainder, and ambulation from an independent broad-based gait to lifelong non-ambulation. Two recurrent-variant carriers (Sommerville 2017 and Ozaki 2020) [9,12] had no detectable enamel defect and three had a normal brain MRI, so neither hallmark is obligate and their absence should not exclude the diagnosis. No modifier explaining this variability has been identified, and the published cohort is far too small for formal analysis; prospectively ascertained cohorts with uniform phenotyping will be required before correlations beyond the seizure and skeletal signals described above can be regarded as established.

#### 3.2.7. Diagnostic Approach

**Clinical suspicion:** HADDTS should be suspected in any infant or child with global developmental delay, significant hypotonia, progressive or static cerebellar ataxia, and tooth enamel abnormalities without an alternative diagnosis [2,17,18]. It may masquerade as primary CFTD when muscular features predominate (Ozaki 2020) or as primary mitochondrial disease (Sommerville 2017) [9,12]. Dysmorphism is minimal to absent in classic HADDTS; its presence suggests alternative or atypical *CTBP1* variants, as in the Zhang (2024) case [21].

**Genetic testing:** Whole-exome sequencing has been the primary diagnostic modality, with whole-genome sequencing used in the Marco (2025) case for improved detection of intronic and regulatory variants [2,9,11,12,13,14,15,16,17,18,19,20,21]. All identified variants require Sanger confirmation and parental segregation [34] to establish de novo status, and ClinVar submission of novel variants is strongly encouraged [2,13,17].

**Mitochondrial and muscle analysis:** Systematic evaluation of mitochondrial function, respiratory chain enzyme activities and citrate synthase in muscle, blood and CSF lactate, and lactate-to-pyruvate ratio, is warranted given the documented overlap and its therapeutic implications [9,16,18]. Muscle biopsy with comprehensive fibre typing and electron microscopy should be performed early, as CFTD may be an initial presentation [12,18]. Respirometry in PBMCs (Seahorse XFp Mito Stress Test) is a useful, less invasive complementary modality, particularly for serial monitoring of bioenergetic response to cofactor supplementation when repeat biopsy is impractical [20].

### 3.3. Management

No disease-modifying therapies exist as of July 2026; management is supportive, symptomatic, and multidisciplinary [2,9,17,18]. Neurology provides seizure surveillance, neurodevelopmental monitoring, and rehabilitation; nutrition and gastroenterology address dysphagia and failure to thrive, including gastrostomy where indicated; pulmonology manages aspiration risk and respiratory support; dentistry provides early evaluation, protective restorations, and caries prevention; speech and language pathology supports AAC and swallowing; and orthopaedics manages scoliosis, mobility, and contracture prevention [2,13,14,15,17,18]. Mitochondrial cofactor supplementation (coenzyme Q10, riboflavin, L-carnitine, ascorbic acid) should be considered when respiratory chain dysfunction is confirmed: Wong et al. (2022) reported subjective improvement, and Ivanov et al. (2026) provided objective, longitudinal evidence of partial bioenergetic recovery without motor, language, or cognitive gain [16,20]. This dissociation indicates that cofactor supplementation can ameliorate the secondary mitochondrial component but is unlikely to modify the underlying CTBP1-driven trajectory and should not be presented to families as disease-modifying.

### 3.4. Emerging Therapies

No disease-modifying therapies are currently available or in active clinical development for HADDTS. Akdas et al. (2023) generated CRISPR/Cas9-engineered hESC lines carrying allele-specific *CTBP1* variants, providing isogenic platforms for drug screening in which mutant and wild-type cells are genetically matched and confounding genetic background is eliminated [35]. Vijayalingam et al. (2020) and Lee et al. (2025) further established both engineered and patient-derived iPSC models, offering complementary systems for functional studies and preclinical screening [3,31]. The observation that mutant CTBP1 can interfere with CTBP2 through heterodimerisation [17] indicates that therapeutic strategies may need to address not only restoration of CTBP1 function but also mitigation of trans-paralog interference with CTBP2-dependent programmes. In this context, allele-specific antisense oligonucleotide (ASO) approaches selectively targeting the mutant transcript represent a plausible strategy, as they could reduce production of the dominant-negative protein while preserving wild-type CTBP1 and CTBP2 function; however, such approaches remain conceptual and have not yet entered preclinical development.

### 3.5. Non-Peer-Reviewed and Unpublished Cases

The 25 peer-reviewed patients (Table 2) represent only a fraction of individuals with confirmed *CTBP1* variants, as evidenced by four independent, verifiable sources: ClinVar submissions, DECIPHER records, conference abstracts, and patient registries. ClinVar (Variation ID 225758; c.991C>T) has received 13 germline pathogenic submissions from independent clinical and research laboratories as of July 2026, 12 contributing to the aggregate “Pathogenic” classification; several originate from institutions that have never published HADDTS case reports, including the New York Genome Center, Revvity Omics, Illumina Laboratory Services, the Institute of Human Genetics at FAU Erlangen-Nürnberg, 3billion Inc., and the Broad Institute/GREGoR Consortium, confirming the existence of genetically verified but unpublished patients (Supplementary Table S2). An oral abstract by Gezdirici (2019) described a Turkish patient identified by whole-exome-sequencing re-analysis, distinct from the later published Turkish case of Sunnetci-Akkoyunlu et al. (2025) [19,36]. The Simons Searchlight platform maintains an active CTBP1-related disorder registry; as of 2026 it acknowledges at least 25 individuals described in the literature, with 24 having approved laboratory reports and 17 with completed medical history data, providing critical infrastructure for natural history studies [33]. The HADDTS Foundation reports awareness, as of 24 May 2026, of 50 reported individuals carrying a *CTBP1* mutation, including four who died before age 19 within the preceding four years, and of 20 families registered directly, of whom nine have Foundation-verified genetic reports (Supplementary Document S2) [23]. Because overlap between the Simons Searchlight and Foundation cohorts is unknown, these figures should not be interpreted as independent additive counts; nevertheless they indicate that the known case burden substantially exceeds the 25 peer-reviewed cases, reflecting both under-ascertainment and the recent increase in identification following establishment of the Foundation.

DECIPHER was interrogated as a fourth source. Three open-access patient records carry a *CTBP1* sequence variant (patients 263727, 303970 and 428029; Supplementary Table S2), and all three carry the same recurrent heterozygous missense change at the *CTBP1* locus, reported as c.1024C>T; p.Arg342Trp on ENST00000290921.10 in two records and as c.991C>T; p.Arg331Trp on NM_001012614.2 in the third. Each is classified Pathogenic with full contribution to phenotype by the depositing centre, under ACMG criteria PM2, PS1, PS2 and PP3 (263727), PM2, PS1, PM1, PM6 and PP4 (303970), and PM2, PS1, PS3, PP3 and PM6 (428029); none are present in gnomAD. Inheritance is recorded as de novo with confirmed parentage in patient 263727 and as unknown in the other two. The reported phenotypes are concordant with the HADDTS spectrum: cerebellar hypoplasia or atrophy in all three, with developmental regression, hyporeflexia, lower limb spasticity and thoracolumbar scoliosis in 263727; ataxia, generalised hypotonia, myopathy, global developmental delay and short stature in 303970; and progressive cerebellar ataxia with chronic axonal neuropathy in 428029. None of the three records carries a linked citation, so whether they represent additional unpublished individuals or entries already among the 25 published patients cannot be established from DECIPHER alone; they are therefore not added to the case count.

Copy-number records at the locus are informative in a different sense. The 448 copy-number variants overlapping *CTBP1* are almost all large terminal 4p16.3 rearrangements involving many contiguous genes, and the individuals carrying them are reported with 4p deletion or duplication phenotypes rather than with the HADDTS tetrad (Supplementary Table S3). Loss of one complete *CTBP1* allele therefore does not reproduce the syndrome, which is concordant with the population tolerance of predicted loss-of-function *CTBP1* alleles in gnomAD v4.1.1 (Supplementary Table S1) and adds independent, population-scale support for a dominant-negative rather than a haploinsufficiency mechanism [17,25]. DECIPHER returned no variant class absent from the published spectrum, in particular no canonical splice-site variant, no in-frame insertion or deletion, and no intragenic deletion, so the five variants in Table 1 represent the complete curated mutational spectrum of *CTBP1*-related neurodevelopmental disease identifiable through these sources as of July 2026.

### 3.6. Discussion

HADDTS is an ultra-rare neurodevelopmental syndrome first described in 2016 and defined by a single recurrent de novo missense hotspot mutation (p.Arg342Trp on NM_001328.2; p.Arg331Trp on NM_001012614.2) accounting for >80% of published cases [2,9,11,12,13,14,15,16,17,18,19,20,21]. Several themes emerge from this synthesis. First, phenotypic variability within the same genotype is substantial: the identical p.Arg342Trp (NM_001328.2) variant produces a spectrum ranging from near-normal cognition (Beck 2016 Patient 3) to profound disability with non-ambulation and developmental regression (Beck 2016 Patients 1–2; Sommerville 2017; Ozaki 2020), likely reflecting genetic-background modifiers, epigenetic regulation, and stochastic developmental effects, the systematic characterisation of which will require larger cohorts [2,9,12]. Second, HADDTS is a neurodevelopmental-mitochondrial overlap syndrome in a clinically significant subset, with respiratory chain dysfunction documented biochemically, histochemically, and ultrastructurally across four independent publications, and CFTD on muscle biopsy at age 4 demonstrating that muscle pathology may precede genetic diagnosis by years; given the small number of patients undergoing formal mitochondrial evaluation, the true prevalence of this overlap is almost certainly underestimated [2,9,11,12,13,14,15,16,17,18,19,20,21].

Third, the demonstration that mutant CTBP1 can heterodimerise with wild-type CTBP2 [17] represents a critical mechanistic insight. Because *Ctbp2* homozygous knockout is embryonic lethal in mouse [24], even partial functional compromise of CTBP2 through poisoned CTBP1–CTBP2 heterodimers could amplify phenotypic severity beyond what CTBP1 disruption alone would produce. This trans-paralog dominant-negative model explains several otherwise puzzling features of HADDTS: the devastating severity despite tolerance of CTBP1 haploinsufficiency in the general population [17,25]; the multisystem phenotype affecting neural, muscular, dental, and mitochondrial compartments [2,9,12,17,18]; and the potential for a progressive or neurodegenerative component in a subset [9,12,17]. Future functional studies should directly test whether the CTBP1–CTBP2 heterodimer formation is necessary for disease pathogenesis, as this would have profound implications for therapeutic strategy.

Fourth, the molecular spectrum is expanding beyond the hotspot. The Jafari Khamirani (2021) frameshift in the same PXDLS domain recapitulates most of the classic HADDTS phenotype [13], whereas three non-recurrent variants define an atypical *CTBP1*-related spectrum that diverges from classic HADDTS. The p.Ser124Phe (NM_001328.2; p.Ser113Phe on NM_001012614.2) variant (Zhang 2024), the first reported outside the canonical PXDLS interface, lies in the NAD(H)-binding subdomain, where Ser124 contributes to the cofactor-binding pocket; substitution with phenylalanine is predicted to perturb NAD(H) coordination and the redox-responsive switch governing dimerisation and corepressor activity [21,37]. Clinically the patient diverged from classic HADDTS, with absent enamel defects, non-dominant ataxia, and prominent dysmorphism and pectus excavatum, indicating that disruption of the NAD(H)-binding subdomain produces a mechanistically distinct phenotype [21].

The p.Arg36Pro (NM_001328.2; p.Arg25Pro on NM_001012614.2) variant (Nishijo 2026) illustrates that even variants within the PXDLS cleft can produce a phenotype not fulfilling HADDTS criteria: located in exon 3, structurally remote from Arg342 and engaging a subset of PXDLS-motif cofactors, it drove severe intellectual disability, an atrial septal defect, and dysmorphism without the classic ataxia–enamel–hypotonia tetrad [11]. The p.Ser335Ala (NM_001328.2; p.Ser324Ala on NM_001012614.2) variant (Sunnetci-Akkoyunlu 2025) localises at the boundary between the substrate-binding subdomain and the α5-helix that frames the PXDLS cleft, producing a hybrid phenotype: refractory infantile spasms with hypsarrhythmia (West syndrome), myoclonus, severe hypotonia, feeding difficulties, enamel hypoplasia, and pectus excavatum, accompanied by pachygyria, a thin corpus callosum, and a hypoplastic inferior cerebellar vermis [19]. Three features warrant emphasis: West syndrome had not been reported in any previously published patient, expanding the epilepsy phenotype beyond the occasional seizures of classic cases; pectus excavatum now appears in two atypical-variant carriers and in none of the 21 published p.Arg342Trp (NM_001328.2) patients; and pachygyria represents the first overt cortical malformation in the spectrum, raising the possibility that variants flanking the α5-helix interfere with CTBP1-dependent programmes governing cortical neurogenesis and migration. Across these three patients the canonical tetrad is incompletely expressed while atypical features predominate, arguing that the precise structural element disrupted, not *CTBP1* disruption per se, shapes presentation, analogous to genotype–phenotype relationships in other transcription-factor genes [11,19,21].

Fifth, identification of *CTBP1*’s highest brain expression in the upper rhombic lip, the cerebellar granule-cell progenitor zone, provides neuroanatomical grounding for the cerebellar phenotype and guides iPSC-based cerebellar-organoid modelling, validated molecularly by downregulation of *OLIG3* and *BARHL1* in mutant neurons [3,31,32]. Sixth, the Ivanov et al. (2026) report extends the mitochondrial-overlap concept by quantifying the bioenergetic defect in PBMCs, removing the need for muscle biopsy and providing a longitudinally repeatable readout, while the concurrent ECAR reduction shows that metabolic dysfunction extends beyond OXPHOS to glycolysis; the dissociation between near-complete bioenergetic rescue and absent neurological gain delineates a clear therapeutic ceiling and indicates that disease-modifying intervention must target the mutant transcript or its trans-paralog interference, possibly within an early developmental window [20].

## 4. Conclusions

HADDTS is an ultra-rare but increasingly recognised autosomal dominant neurodevelopmental-mitochondrial syndrome caused by predominantly de novo *CTBP1* variants, with a single recurrent hotspot (p.Arg342Trp on NM_001328.2; p.Arg331Trp on NM_001012614.2) accounting for the large majority of published cases (21 of 25, 84%). The dominant-negative pathomechanism deranges cerebellar neurodevelopmental gene programmes and, in a significant subset, produces secondary mitochondrial dysfunction; the ability of mutant CTBP1 to heterodimerise with and interfere with the essential paralog CTBP2 provides a compelling explanation for the severity and multisystem nature of the phenotype. The phenotypic spectrum ranges from profound neurodisability with regression to relatively mild impairment within the same genotype [2,9,11,12,13,14,15,16,17,18,19,20,21].

Beyond the hotspot, four further variants complete a currently reported spectrum of five distinct *CTBP1* variants. The two-nucleotide frameshift c.1282_1283delCA; p.Gln428ValfsTer84 (NM_001012614.2; reported as c.1315_1316delCA; p.Gln439ValfsTer84 on NM_001328.2) introduces a premature termination codon in the same C-terminal PXDLS domain that harbours the recurrent codon and reproduces most classic HADDTS features, hypotonia being the notable exception, indicating that lesions of this interface converge on a single phenotype irrespective of variant class [13]. The remaining three, p.Ser124Phe, p.Arg36Pro, and p.Ser335Ala (NM_001328.2; p.Ser113Phe, p.Arg25Pro and p.Ser324Ala on NM_001012614.2), define an emerging atypical *CTBP1*-related spectrum in which the canonical tetrad is incompletely expressed and features rarely or never seen in classic HADDTS (West syndrome, dysmorphism, congenital heart disease, pectus excavatum, cortical malformation) can predominate [11,19,21]. Three observations have practical implications. First, the three atypical variants share a positional pattern: each lies outside the recurrent Arg342 codon but within or adjacent to the structural surfaces (PXDLS cleft, α5-helix, NAD(H)-binding pocket) that govern cofactor recruitment and dimerisation, suggesting that the precise element disrupted shapes presentation. Second, pectus excavatum has now been documented in two atypical-variant patients (p.Ser124Phe and p.Ser335Ala, both on NM_001328.2) and in none of the 21 published p.Arg342Trp (NM_001328.2) carriers, raising the possibility that skeletal involvement is a recurring feature of non-canonical variants. Third, the appearance of West syndrome, pachygyria, congenital heart disease, and dysmorphism across these cases argues that severe epileptic encephalopathy, structural cortical anomalies, and skeletal features should be systematically ascertained in future *CTBP1* cohorts. Recognising this broader spectrum is critical for accurate genotype–phenotype counselling, for selecting cases for functional follow-up, and for designing variant-stratified natural history studies. Key priorities for the field are: (**i**) an international patient registry with prospective longitudinal data; (**ii**) systematic mitochondrial investigation in all diagnosed patients; (**iii**) natural history studies defining progression, respiratory trajectory, and survival; (**iv**) functional characterisation of non-p.Arg342Trp (NM_001328.2) variants and direct testing of the CTBP1–CTBP2 heterodimer-poisoning hypothesis; and (**v**) preclinical development of allele-specific antisense and small-molecule therapeutics targeting the mutant *CTBP1* transcript.

## Figures and Tables

**Table 1:** Confirmed and likely pathogenic *CTBP1* variants. Variants are given on both reference sequences: first the long isoform NM_001328.2, under which most were originally reported, and then the MANE Select transcript NM_001012614.2, which is the recommended reference. Exon numbering follows the MANE Select transcript NM_001012614.2. dbSNP identifiers are given where one has been assigned: the recurrent variant is rs869320802 (ClinVar Variation ID 225758).

cDNA Variant/Protein	Type	Exon/Domain	Pts	ACMG Class	Effect	Ref
c.1024C>T *p.Arg342Trp* (NM_001328.2); c.991C>T *p.Arg331Trp* (MANE Select NM_001012614.2)	Missense	Exon 9/α5-helix, PXDLS cleft	21	Pathogenic	Single amino acid substitution; dominant-negative	[2,9,11,12,14,15,16,17,18,20,22]
c.1315_1316delCA *p.Gln439ValfsTer84* (NM_001328.2); c.1282_1283delCA *p.Gln428ValfsTer84* (MANE Select NM_001012614.2)	Frameshift	Exon 10/C-terminal PXDLS	1	Pathogenic	Premature stop codon; truncated protein, atypical phenotype	[13]
c.371C>T *p.Ser124Phe* (NM_001328.2); c.338C>T *p.Ser113Phe* (MANE Select NM_001012614.2)	Missense	Exon 5/NAD(H)-binding	1	Likely Path.	Single amino acid substitution; NAD(H) domain; atypical phenotype	[21]
c.107G>C *p.Arg36Pro* (NM_001328.2); c.74G>C *p.Arg25Pro* (MANE Select NM_001012614.2)	Missense	Exon 3/PXDLS binding cleft	1	Pathogenic	Single amino acid substitution; PXDLS cleft; atypical phenotype	[11]
c.1003T>G *p.Ser335Ala* (NM_001328.2); c.970T>G *p.Ser324Ala* (MANE Select NM_001012614.2)	Missense	Exon 8/NAD(H)-binding (substrate-binding); adjacent to α5-helix/PXDLS cleft	1	Likely Path.	Single amino acid substitution; substrate-binding subdomain bordering PXDLS cleft; atypical phenotype with West syndrome and cortical malformation	[19]

ACMG = American College of Medical Genetics and Genomics; Pts = patients.

**Table 2:** All peer-reviewed published patients with *CTBP1* pathogenic variants.

#	Year	Sex	Age	cDNA Variant (NM_001328.2)	Protein	Type	Inheritance	Country	Ref
**1**	2016	M	8 y	c.1024C>T	p.Arg342Trp	Mis	de novo (mat. mosaic)	USA	[2]
**2**	2016	M	20 y	c.1024C>T	p.Arg342Trp	Mis	de novo	USA	[2]
**3**	2016	F	9 y	c.1024C>T	p.Arg342Trp	Mis	de novo	USA	[2]
**4**	2016	F	12 y	c.1024C>T	p.Arg342Trp	Mis	de novo	USA	[2]
**5**	2017	F	16 y	c.1024C>T	p.Arg342Trp	Mis	de novo	UK	[9]
**6**	2019	M	20 y	c.1024C>T	p.Arg342Trp	Mis	de novo	NR	[17]
**7**	2019	F	22 y	c.1024C>T	p.Arg342Trp	Mis	de novo	NR	[17]
**8**	2019	M	6 y	c.1024C>T	p.Arg342Trp	Mis	de novo	NR	[17]
**9**	2019	M	6 y	c.1024C>T	p.Arg342Trp	Mis	de novo	NR	[17]
**10**	2019	M	10 y	c.1024C>T	p.Arg342Trp	Mis	de novo	NR	[17]
**11**	2019	M	5 y	c.1024C>T	p.Arg342Trp	Mis	de novo	NR	[17]
**12**	2019	M	11 y	c.1024C>T	p.Arg342Trp	Mis	de novo	NR	[17]
**13**	2020	M	7 y	c.1024C>T	p.Arg342Trp	Mis	de novo	India	[14]
**14**	2020	M	14 y	c.1024C>T	p.Arg342Trp	Mis	de novo	Japan	[12]
**15**	2021	M	25 y	c.1315_1316delCA	p.Gln439ValfsTer84	FS	de novo	Iran	[13]
**16**	2022	F	6 y	c.1024C>T	p.Arg342Trp	Mis	de novo	Australia	[16]
**17**	2023	M	Child	c.1024C>T	p.Arg342Trp	Mis	de novo	Belgium	[18]
**18**	2024	F	Child	c.371C>T	p.Ser124Phe	Mis	de novo	China	[21]
**19**	2025	F	3 y	c.1024C>T	p.Arg342Trp	Mis	de novo	UK	[15]
**20**	2026	M	20 y	c.107G>C	p.Arg36Pro	Mis	de novo	Japan	[11]
**21**	2026	NR	NR	c.1024C>T	p.Arg342Trp	Mis	de novo	Japan	[11]
**22**	2026	NR	NR	c.1024C>T	p.Arg342Trp	Mis	de novo	Japan	[11]
**23**	2025	M	1 y	c.1003T>G	p.Ser335Ala	Mis	de novo	Turkey	[19]
**24**	2026	F	10 y	c.1024C>T	p.Arg342Trp	Mis	de novo	Bulgaria	[20]
**25**	2026	M	7 y	c.1024C>T	p.Arg342Trp	Mis	de novo	Poland	[22]

Variants are listed on the long isoform NM_001328.2, the transcript used in most of the source publications; the corresponding MANE Select (NM_001012614.2) designations are given in Table 1. The recurrent c.1024C>T; p.Arg342Trp (NM_001328.2) is equivalent to c.991C>T; p.Arg331Trp (NM_001012614.2). Mis = missense; FS = frameshift; mat. = maternal; NR = not reported. Ages are at last reported evaluation.

**Table 3:** Patient-by-feature evidence grid (25 patients × 15 features). Variant shorthand in the Source column refers to NM_001328.2 designations (R342W = p.Arg342Trp, S124F = p.Ser124Phe, R36P = p.Arg36Pro, S335Ala = p.Ser335Ala); the corresponding MANE Select (NM_001012614.2) designations are given in Table 1.

Pt	Source (Variant)	F1	F2	F3	F4	F5	F6	F7	F8	F9	F10	F11	F12	F13	F14	F15
**#1**	Beck 2016 P1 (R342W)	+	+	+	+	+	+	+	+	?	-	+	?	?	-	?
**#2**	Beck 2016 P2 (R342W)	+	+	+	+	+	+	+	+	?	-	+	?	+	-	?
**#3**	Beck 2016 P3 (R342W)	+	+	+	-	+	+	-	+	?	-	+	?	+	-	?
**#4**	Beck 2016 P4 (R342W)	+	+	+	+	+	+	+	-	?	- *	+	?	+	-	?
**#5**	Sommerville (R342W)	+	+	+	+	+	-	+	+	+	+	+	+	?	-	?
**#6**	Beck 2019 P5 (R342W)	+	+	+	+	+	?	?	?	?	-	+	?	?	-	?
**#7**	Beck 2019 P6 (R342W)	+	+	+	+	+	+	?	+	?	+	-	?	+	-	?
**#8**	Beck 2019 P7 (R342W)	+	+	+	+	+	+	?	-	?	-	-	?	?	-	?
**#9**	Beck 2019 P8 (R342W)	+	+	+	+	+	+	?	+	?	-	+	?	?	-	?
**#10**	Beck 2019 P9 (R342W)	+	+	+	+	+	+	?	+	?	+	-	?	?	-	?
**#11**	Beck 2019 P10 (R342W)	+	+	+	+	+	?	?	+	?	+	-	?	-	-	?
**#12**	Beck 2019 P11 (R342W)	+	+	+	+	+	+	?	+	?	-	-	?	+	-	?
**#13**	Bhatia (R342W)	+	+	+	+	+	+	+	+	+	-	+	?	?	-	?
**#14**	Ozaki (R342W)	+	+	+	+	+	-	+	+	+	+	+	+	?	-	?
**#15**	Khamirani (frameshift)	+	-	+	+	-	+	-	?	?	-	-	?	-	+	+
**#16**	Wong (R342W)	+	+	+	+	+	+	+	+	-	-	+	+	-	-	?
**#17**	Kadhim (R342W)	+	+	+	+	+	+	+	+	+	-	+	+	-	-	?
**#18**	Zhang (S124F)	+	-	+	+	-	-	+	-	?	-	-	?	?	-	+
**#19**	Marco (R342W)	+	+	+	+	+	+	+	+	+	+	+	?	+	-	?
**#20**	Nishijo R36P #1	+	-	+	+	?	-	+	?	?	?	?	?	?	-	+
**#21**	Nishijo R342W #2	+	+	+	+	?	+	+	+	?	+	+	?	+	?	?
**#22**	Nishijo R342W #3	+	+	+	+	?	+	-	-	?	+	+	?	-	?	?
**#23**	Sunnetci (S335Ala)	+	+	+	+	?	+	+	+	?	?	?	?	?	+	+
**#24**	Ivanov (R342W)	+	+	+	+	+	+	+	+	+	+	+	+	-	-	?
**#25**	Jedrzejowska (R342W)	+	+	+	+	+	+	+	+	?	+	?	?	?	-	-

+ documented present; - documented absent/normal; ? not reported/no individual data. F1 Global developmental delay; F2 hypotonia; F3 language/dysarthria; F4 intellectual disability; F5 ataxia; F6 tooth enamel; F7 feeding/FTT; F8 cerebellar atrophy (MRI); F9 scoliosis; F10 regression; F11 myopathy; F12 mitochondrial dysfunction; F13 oculomotor apraxia; F14 seizures; F15 pectus/skeletal. * Beck et al. (2019) tabulated 12 individuals, the four originally reported by Beck et al. (2016) (their patients 1–4), the Sommerville et al. (2017) patient (their patient 12), and seven newly reported individuals (their patients 5–11). To avoid double-counting, only the seven new individuals are listed separately here (#6–#12).

**Table 4:** Clinical feature frequency and severity in HADDTS (n = 25, 21 patients with the recurrent p.Arg342Trp/p.Arg331Trp (NM_001328.2/NM_001012614.2) variant and four with non-recurrent variants).

Clinical Feature	Frequency	Severity Range	Notes
**Global developmental delay**	25/25	Moderate-Severe	Universal feature across all variants; involves motor, language and cognitive domains; earliest presenting concern in most cases.
**Hypotonia**	22/25	Mild to severe; axial and appendicular	Explicitly absent in the atypical-variant patients of Jafari Khamirani (p.Gln439ValfsTer84), Zhang (p.Ser124Phe) and the Nishijo p.Arg36Pro proband; severe hypotonia is documented in Sunnetci-Akkoyunlu (p.Ser335Ala). Ivanov initially had normal tone in infancy with progressive decline.
**Language delay/dysarthria**	25/25	Moderate-Severe	Universal language delay; many patients nonverbal or AAC-dependent. Explicit dysarthria described in Beck 2016 Patients 1–3, Beck 2019 Patients 7, 10, 11, Bhatia, Khamirani, and Marco; Ivanov lost previously acquired words during regression.
**Intellectual disability**	24/25	Moderate-Severe	Sole documented exception: Beck 2016 Patient 3 (F, 9 y) with age-appropriate cognition and functional speech.
**Ataxia/cerebellar dysfunction**	19/25	Ataxic gait to non-ambulatory	Explicitly absent in Zhang (p.Ser124Phe); not documented in Sunnetci-Akkoyunlu (p.Ser335Ala, 1-year-old, pre-ambulatory). Only partial (“+/−”) in Khamirani frameshift case, recorded here as absent. Nishijo documents ataxia-like behaviours but not explicitly ataxia.
**Tooth enamel defects**	19/25	Hypoplastic, soft, discoloured; root resorption in some	Beck 2019 documents 9 of 12 explicit cases (1 explicitly absent and 2 not described). Explicitly absent in Sommerville and Ozaki despite recurrent p.Arg342Trp; absent in Zhang (p.Ser124Phe). Ivanov: “dystrophic teeth” documented in full text. Not described in Beck 2019 Patients 5 and 10.
**Feeding difficulties/FTT**	15/25	Tube feeding in many	Documented in Beck 2016 Patients 1, 2 and 4 (explicitly absent in Patient 3), Sommerville, Bhatia, Wong (failure to thrive), Kadhim, Marco, Zhang, and Ozaki (severe non-ambulatory disease). Beck 2019 Patient 6 inferred from bulbar weakness and microcephaly. Ivanov: gastrostomy placement at age 9 y with weight below 3rd centile. Khamirani explicitly normal weight.
**Cerebellar atrophy (MRI)**	18/25	Mild volume loss to severe; occasionally progressive	Normal MRI in Beck 2016 Patient 4, Beck 2019 Patient 7, Zhang, and Nishijo patient 3. Progression documented on serial imaging in Ozaki and Beck 2019 Patients 2 and 6. Subtle findings (prominent foliae, mild folia hypoplasia) in Bhatia and Marco. Ivanov: cerebellar atrophy at age 6 y. MRI not performed or not reported in 3 cases.
**Scoliosis**	6/25	Mild to severe; secondary to axial hypotonia/non-ambulation; surgical correction in severe cases	Explicitly documented in Sommerville (with contractures), Bhatia (with limb contractures), Marco (mild), and Ivanov (severe kyphoscoliosis requiring surgical correction at 8 y). Additional secondary scoliosis in non-ambulatory or severely myopathic patients reported by Beck 2016 (non-ambulatory P2), Beck 2019 (Patient 6 with multiple contractures), Ozaki (progressive non-ambulation), and Kadhim (dystrophic myopathy with skeletal anomalies). Not present in Zhang (p.Ser124Phe), whose skeletal phenotype features pectus excavatum and radial-head dislocation instead.
**Developmental regression**	10/25	Motor and/or language loss	Beck 2019 documents 4 patients (Patient 6: motor + language; Patient 9: motor + cognitive; Patient 10: motor; Sommerville/Patient 12: motor + language). Plus Ozaki (severe psychomotor regression with progressive cerebellar atrophy), Marco (lost ambulation at 5 y), and Ivanov (lost walking at 6 y; lost words, pointing, sitting and rolling by 9 y). Nishijo patient 2 (slow regression of motor/swallowing) and patient 3 (regression of motor and language).
**Muscle weakness/myopathy**	15/25	Fibre-size variability, CFTD, dystrophic, vacuolar, with mitochondrial features	Histologically documented in Beck 2016 (4 patients), Sommerville, Bhatia, Ozaki (CFTD), Wong, Kadhim, and Marco (centronuclear myopathy); EMG-only myopathic pattern in Beck 2019 Patients 5 and 8. Ivanov: clinical myopathy with generalised muscle hypotrophy and multiple flexion contractures, no biopsy documented. Beck 2019 Patient 6 had ulnar mononeuropathies rather than primary myopathy. Nishijo patients both have biopsy-proven congenital fibre-type disproportion.
**Mitochondrial dysfunction**	5/25	Complexes I and IV decreased; reduced PBMC OCR and ECAR	Likely underdiagnosed; systematic evaluation warranted. Kadhim and Ozaki are only structural.
**Oculomotor apraxia**	7/25	Mild to moderate	Explicitly described in 5 of 12 Beck 2019 patients, plus Marco and Nishijo patient 2. Ivanov shows exotropia and hyperactive oculocephalic reflex, but these are not classified as oculomotor apraxia proper.
**Seizures/epilepsy**	2/25	Variable	Khamirani frameshift: one myoclonic seizure at age 5 y. Sunnetci-Akkoyunlu p.Ser335Ala: West syndrome in 1-year-old male. Beck 2019 explicitly reported no seizures in 12 patients.
**Pectus excavatum/skeletal anomalies**	4/25	Variable	Zhang p.Ser124Phe: microcephaly, pectus excavatum, congenital radial-head dislocation, single palmar crease, short 5th finger, synophrys. Khamirani frameshift: frontal bossing, deep-set eyes. Sunnetci-Akkoyunlu p.Ser335Ala (NM_001328.2): pectus excavatum. Nishijo p.Arg36Pro: atrial septal defect and dysmorphic features. Minor non-classifying dysmorphism in some classic R342W patients (e.g., Beck 2016 P2: frontal bossing/deep-set eyes; Marco: blue sclerae, syndactyly) is not counted here.

Refs [2,9,11,12,13,14,15,16,17,18,19,20,21,22]. CFTD = congenital fibre-type disproportion; AAC = augmentative and alternative communication; FTT = failure to thrive. Variant designations in the Notes column are given on NM_001328.2; the corresponding MANE Select (NM_001012614.2) designations are given in Table 1.

## Data Availability

All data analysed in this review are available within the cited primary publications and in publicly accessible databases, including PubMed, ClinVar (Variation ID 225758), DECIPHER, OMIM (#617915), the HADDTS Foundation, DECIPHER, and gnomAD v4.1.1. No new datasets were generated or analysed.

## Supplementary Materials

The following supporting information can be downloaded at: https://www.mdpi.com/article/10.3390/ijms27157065/s1.

## References

[1] Akdaş E.Y., Schneider K., Lu D., Hachani K., Roberts E.L., Ghanem M., Dezfouli A.B., Wollenberg B. (2026). NAD(H)-dependent corepressor CTBP1 integrates metabolic signals to drive oncogenic programs. Front. Immunol..

[2] Beck D.B., Cho M.T., Millan F., Yates C., Hannibal M., O’cOnnor B., Shinawi M., Connolly A.M., Waggoner D., Halbach S. (2016). A recurrent de novo CTBP1 mutation is associated with developmental delay, hypotonia, ataxia, and tooth enamel defects. Neurogenetics.

[3] Lee S., Vijayalingam S., Klotz E., Dedert C., Xu F., Chinnadurai G., Ezekiel U.R. (2025). Isogenic iPSC-derived CTBP1 mutant neuronal cells exhibit neurodevelopmental defects. Front. Neurosci..

[4] Hu K., Li Y., Yu H., Hu Y. (2019). CTBP1 confers protection for hippocampal and cortical neurons in rat models of Alzheimer’s disease. Neuroimmunomodulation.

[5] Raza U., Saatci Ö., Uhlmann S., Ansari S.A., Eyüpoğlu E., Yurdusev E., Mutlu M., Ersan P.G., Altundağ M.K., Zhang J.D. (2016). The miR-644a/CTBP1/p53 axis suppresses drug resistance by simultaneous inhibition of cell survival and epithelial-mesenchymal transition in breast cancer. Oncotarget.

[6] Arthur S.A., Blaydes J.P., Houghton F.D. (2019). Glycolysis Regulates Human Embryonic Stem Cell Self-Renewal under Hypoxia through HIF-2α and the Glycolytic Sensors CTBPs. Stem Cell Rep..

[7] Cattaneo O., Lopez G., Rajendran J., Chabry F., Liaudet N., Startchik S., Prola A., Castets P. (2026). CtBP1 sustains activity-dependent muscle properties and dampens synaptic, contractile and metabolic changes triggered by denervation. Skelet. Muscle.

[8] Kim J., Youn H. (2009). C-terminal binding protein maintains mitochondrial activities. Cell Death Differ..

[9] Sommerville E.W., Alston C.L., Pyle A., He L., Falkous G., Naismith K., Chinnery P.F., McFarland R., Taylor R.W. (2017). De novo CTBP1 variant is associated with decreased mitochondrial respiratory chain activities. Neurol. Genet..

[10] Gastaldi L., Martín J.I., Sosa L.J., Quassollo G., Cuasolo Y.M.P., Valente C., Luini A., Corda D., Cáceres A., Bisbal M. (2022). BARS influences neuronal development by regulation of post-golgi trafficking. Cells.

[11] Nishijo T., Yanagi K., Ito H., Hamada N., Nakamura S., Chinen Y., Fukuhara Y., Iwamoto I., Kaname T., Okamoto N. (2026). CTBP1 in Brain Development: A Novel Variant c. 107G> C, p.(R36P) Leads to a Distinct Neurodevelopmental Disorder. J. Neurochem..

[12] Ozaki A., Komaki H., Nishino I., Nonaka I., Ikuta Y., Sakamoto M., Iwama K., Mizuguchi T., Matsumoto N., Sasaki M. (2020). Developmental regression and cerebellar atrophy in a patient with congenital fiber-type disproportion and a de novo heterozygous CTBP1 variant. No To Hattatsu.

[13] Khamirani H.J., Zoghi S., Sichani A.S., Dianatpour M., Mohammadi S., Tabei S.M.B., Dastgheib S.A. (2021). Exome sequencing identified a de novo frameshift pathogenic variant of CTBP1 in an extremely rare case of HADDTS. J. Genet..

[14] Bhatia S.K., Arora V., Verma I.C. (2020). A further case of hypotonia, ataxia, developmental delay and tooth enamel defect syndrome due to a recurrent C-terminal binding protein 1 mutation. Clin. Dysmorphol..

[15] Marco S.B.S., Pardington E., Monaghan M., Spaull R., Fadilah A., Kurian K., Vijayakumar K., Smithson S., Majumdar A. (2025). Hypotonia, Ataxia, Developmental Delay and Tooth Enamel Defect Syndrome (HADDTS) due to a Heterozygous de Novo Missense Variant in CTBP1 Identified via Whole Genome Sequencing. Case Rep. Pediatr..

[16] Wong W., Balasubramaniam S., Wong R.S.H., Graf N., Thorburn D.R., McFarland R., Troedson C. (2022). Mitochondrial respiratory chain dysfunction in a patient with a heterozygous de novo CTBP1 variant. JIMD Rep..

[17] Beck D.B., Subramanian T., Vijayalingam S., Ezekiel U.R., Donkervoort S., Yang M.L., Dubbs H.A., Ortiz-Gonzalez X.R., Lakhani S., Segal D. (2019). A pathogenic CtBP1 missense mutation causes altered cofactor binding and transcriptional activity. Neurogenetics.

[18] Kadhim H., El-Howayek E., Coppens S., Duff J., Topf A., Kaleeta J.-P., Simoni P., Boitsios G., Remiche G., Straub V. (2023). A pathogenic CTBP1 variant featuring HADDTS with dystrophic myopathology. Neuromuscul. Disord..

[19] Sunnetci-Akkoyunlu D., Kara B., Ozer T., Deniz A., Sakarya-Gunes A., Isik E.B., Dogruoglu B., Ilkay Z., Yilmaz M., Sahin S. (2025). Genetic Etiology of Developmental and Epileptic Encephalopathy in a Turkish Cohort: A Single-Center Study with Targeted Gene Panel and Whole Exome Sequencing. Genes.

[20] Ivanov Z., Gevezova M., Pacheva I., Ketev K., Chochkova-Bukova L., Sarafian V., Ivanov I. (2026). A Rare CTBP1-Related Neurodevelopmental Disorder Is Associated with Impaired Mitochondrial Bioenergetics: A Functional Case Report. Int. J. Mol. Sci..

[21] Zhang Q., Liu Y., Liu X., Zhao Y., Zhang J. (2024). A novel CTBP1 variant in a Chinese pediatric patient with a phenotype distinct from hypotonia, ataxia, developmental delay, and tooth enamel defect syndrome. Front. Genet..

[22] Jędrzejowska M., Jurkiewicz E., Gos M., Rokicki D., Madej-Pilarczyk A. (2026). Floppy baby syndrome as the first presentation of HADDTS associated with CTBP1 mutation. Folia Neuropathol..

[23] HADDTS Foundation. https://haddtsfoundation.org.

[24] Hildebrand J.D., Soriano P. (2002). Overlapping and unique roles for C-terminal binding protein 1 (CtBP1) and CtBP2 during mouse development. Mol. Cell. Biol..

[25] Karczewski K., Francioli L. (2017). The Genome Aggregation Database (gnomAD).

[26] Page M.J., McKenzie J.E., Bossuyt P.M., Boutron I., Hoffmann T.C., Mulrow C.D., Shamseer L., Tetzlaff J.M., Akl E., Brennan S.E. (2021). The PRISMA 2020 statement: An updated guideline for reporting systematic reviews. BMJ.

[27] Landrum M.J., Lee J.M., Riley G.R., Jang W., Rubinstein W.S., Church D.M., Maglott D.R. (2014). ClinVar: Public archive of relationships among sequence variation and human phenotype. Nucleic Acids Res..

[28] Foreman J., Brent S., Perrett D., Bevan A.P., Hunt S.E., Cunningham F., Hurles M.E., Firth H.V. (2022). DECIPHER: Supporting the interpretation and sharing of rare disease phenotype-linked variant data to advance diagnosis and research. Hum. Mutat..

[29] Acosta-Baena N., Tejada-Moreno J.A., Arcos-Burgos M., Villegas-Lanau C.A. (2022). CTBP1 and CTBP2 mutations underpinning neurological disorders: A systematic review. Neurogenetics.

[30] Kuppuswamy M., Vijayalingam S., Zhao L.-J., Zhou Y., Subramanian T., Ryerse J., Chinnadurai G. (2008). Role of the PLDLS-binding cleft region of CtBP1 in recruitment of core and auxiliary components of the corepressor complex. Mol. Cell. Biol..

[31] Vijayalingam S., Ezekiel U.R., Xu F., Subramanian T., Geerling E., Hoelscher B., San K., Ganapathy A., Pemberton K., Tycksen E. (2020). Human iPSC-derived neuronal cells from CTBP1-mutated patients reveal altered expression of neurodevelopmental gene networks. Front. Neurosci..

[32] Lee S., Ezekiel U.R. (2025). Using the allen brain cell atlas of the human brain to gain insights into C-Terminal-Binding protein 1 (CtBP1)’s potential function. Biologics.

[33] Simons Searchlight (2025). CTBP1-Related Disorder Registry.

[34] Rubinstein W.S., Maglott D.R., Lee J.M., Kattman B.L., Malheiro A.J., Ovetsky M., Hem V., Gorelenkov V., Song G., Wallin C. (2012). The NIH genetic testing registry: A new, centralized database of genetic tests to enable access to comprehensive information and improve transparency. Nucleic Acids Res..

[35] Akdaş E.Y., Turan S., Guhathakurta D., Ekici A., Salar S., Lie D.C., Winner B., Fejtova A. (2023). CRISPR/Cas9-mediated generation of hESC lines with homozygote and heterozygote p.R331W mutation in CTBP1 to model HADDTS syndrome. Stem Cell Res..

[36] Gezdirici A. (2019). S-15-Diagnosing Process in a Newly Described Extremely Rare Disease by Whole Exome Sequencing Re-analysis. 13th Balkan Congress of Human Genetics.

[37] Nichols J.C., Schiffer C.A., Royer W.E. (2021). NAD(H) phosphates mediate tetramer assembly of human C-terminal binding protein (CtBP). J. Biol. Chem..

