# Supplementary material for: Delineating the CTBP1-Related Phenotypic Spectrum: A Review of HADDTS and Atypical Variants
Source: Int J Mol Sci. 2026 Aug 6;27(15):7065. doi: 10.3390/ijms27157065 (PMC13467200; doi:10.3390/ijms27157065)
Supplement: Supplementary file 1 [file ijms-27-07065-s001.zip › Supplementray document S1.pdf]

PRISMA 2020 Checklist

The Expanding CTBP1 Spectrum: A Systematic Review of HADDTS and Atypical Variants.

This systematic review was reported in accordance with the PRISMA 2020 statement. Because the available evidence consisted of single-patient case reports, small case series, and genotype–phenotype descriptions, findings were synthesised descriptively and no meta-analysis was performed; meta-analysis-specific items are therefore marked "not applicable."

Section and Topic	Item #	Checklist item	Location where item is reported
**TITLE**			
Title	1	Identify the report as a systematic review.	Title page ("The Expanding CTBP1 Spectrum: A Systematic Review of HADDTS and Atypical Variants").
**ABSTRACT**			
Abstract	2	See the PRISMA 2020 for Abstracts checklist.	Abstract.
**INTRODUCTION**			
Rationale	3	Describe the rationale for the review in the context of existing knowledge.	Background.
Objectives	4	Provide an explicit statement of the objective(s) or question(s) the review addresses.	Background (final paragraph); Abstract.
**METHODS**			
Eligibility criteria	5	Specify the inclusion and exclusion criteria for the review and how studies were grouped for the syntheses.	Methods.
Information sources	6	Specify all databases, registers, websites, organisations, reference lists and other sources searched or consulted to identify studies. Specify the date when each source was last searched or consulted.	Methods (PubMed/MEDLINE, Google Scholar, ClinVar, OMIM, DECIPHER, Simons Searchlight, bioRxiv, medRxiv, HADDTS Foundation; from inception through June 2026).
Search strategy	7	Present the full search strategies for all databases, registers and websites, including any filters and limits used.	Methods (search terms and variant designations listed).
Selection process	8	Specify the methods used to decide whether a study met the inclusion criteria of the review, including how many reviewers screened each record and each report retrieved, whether they worked independently, and if applicable, details of automation tools used in the process.	Methods (two reviewers searched and screened independently).
Data collection process	9	Specify the methods used to collect data from reports, including how many reviewers collected data from each report, whether they worked independently, any processes for obtaining or confirming data from study investigators, and if applicable, details of automation tools used in the process.	Methods; Authors' contributions (data extraction by two reviewers).

Section and Topic	Item #	Checklist item	Location where item is reported
Data items	10a	List and define all outcomes for which data were sought. Specify whether all results that were compatible with each outcome domain in each study were sought (e.g. for all measures, time points, analyses), and if not, the methods used to decide which results to collect.	Methods; Clinical Phenotype and Natural History; Tables 3 and 4.
	10b	List and define all other variables for which data were sought (e.g. participant and intervention characteristics, funding sources). Describe any assumptions made about any missing or unclear information.	Methods (demographics, genotype, neuroimaging, muscle pathology, mitochondrial function; missing data handled as variable denominators).
Study risk of bias assessment	11	Specify the methods used to assess risk of bias in the included studies, including details of the tool(s) used, how many reviewers assessed each study and whether they worked independently, and if applicable, details of automation tools used in the process.	Methods. Formal risk-of-bias tools were not applicable because the evidence base comprised single-patient case reports and small case series; limitations are addressed in the Discussion.
Effect measures	12	Specify for each outcome the effect measure(s) (e.g. risk ratio, mean difference) used in the synthesis or presentation of results.	Methods. Not applicable — no quantitative effect measures; findings synthesised descriptively as approximate frequencies (Table 4).
Synthesis methods	13a	Describe the processes used to decide which studies were eligible for each synthesis (e.g. tabulating the study intervention characteristics and comparing against the planned groups for each synthesis (item #5)).	Methods (patients grouped as classic vs. atypical CTBP1-related spectrum).
	13b	Describe any methods required to prepare the data for presentation or synthesis, such as handling of missing summary statistics, or data conversions.	Methods (frequencies expressed as approximate percentages; denominators vary by feature owing to

PRISMA 2020 Checklist

Section and Topic	Item #	Checklist item	Location where item is reported
			incomplete reporting).
	13c	Describe any methods used to tabulate or visually display results of individual studies and syntheses.	Methods; Tables 1–4; Figures 1–2.
	13d	Describe any methods used to synthesize results and provide a rationale for the choice(s). If meta-analysis was performed, describe the model(s), method(s) to identify the presence and extent of statistical heterogeneity, and software package(s) used.	Methods. Narrative (descriptive) synthesis was used; no meta-analysis was performed because the evidence consisted of case reports and small series.
	13e	Describe any methods used to explore possible causes of heterogeneity among study results (e.g. subgroup analysis, meta-regression).	Not applicable — no meta-analysis. Genotype-based subgroups (classic vs. atypical) described narratively in Methods and Results.
	13f	Describe any sensitivity analyses conducted to assess robustness of the synthesized results.	Not applicable — no quantitative synthesis performed.
Reporting bias assessment	14	Describe any methods used to assess risk of bias due to missing results in a synthesis (arising from reporting biases).	Discussion (under-ascertainment and publication/reporting bias addressed); Non-Peer-Reviewed and Unpublished Cases.
Certainty assessment	15	Describe any methods used to assess certainty (or confidence) in the body of evidence for an outcome.	Methods; Discussion. Formal GRADE assessment was not applicable to the case-report evidence base; certainty is discussed narratively.
**RESULTS**			
Study selection	16a	Describe the results of the search and selection process, from the number of records identified in the search to the number of studies included in the review, ideally using a flow diagram.	Methods/Results; Non-Peer-Reviewed

PRISMA

PRISMA 2020 Checklist

Section and Topic	Item #	Checklist item	Location where item is reported
			and Unpublished Cases (25 peer-reviewed cases included).
	16b	Cite studies that might appear to meet the inclusion criteria, but which were excluded, and explain why they were excluded.	Methods (functional/molecular studies without primary patient data and duplicate reports excluded); Non-Peer-Reviewed and Unpublished Cases.
Study characteristics	17	Cite each included study and present its characteristics.	Tables 1–4; Genetics and Clinical Phenotype sections.
Risk of bias in studies	18	Present assessments of risk of bias for each included study.	Not applicable (case-report evidence base); methodological limitations presented in the Discussion.
Results of individual studies	19	For all outcomes, present, for each study: (a) summary statistics for each group (where appropriate) and (b) an effect estimate and its precision (e.g. confidence/credible interval), ideally using structured tables or plots.	Table 2 (all published patients); Table 3 (patient-by-feature evidence grid).
Results of syntheses	20a	For each synthesis, briefly summarise the characteristics and risk of bias among contributing studies.	Clinical Phenotype and Natural History; Methods.
	20b	Present results of all statistical syntheses conducted. If meta-analysis was done, present for each the summary estimate and its precision (e.g. confidence/credible interval) and measures of statistical heterogeneity. If comparing groups, describe the direction of the effect.	Table 4 (clinical feature frequency and severity); Clinical Phenotype and Natural History. No meta-analysis — descriptive frequencies only.
	20c	Present results of all investigations of possible causes of heterogeneity among study results.	Not applicable — no meta-analysis. Phenotypic variability by genotype discussed narratively (Clinical

PRISMA

PRISMA 2020 Checklist

Section and Topic	Item #	Checklist item	Location where item is reported
			Phenotype; Discussion).
	20d	Present results of all sensitivity analyses conducted to assess the robustness of the synthesized results.	Not applicable — no quantitative synthesis.
Reporting biases	21	Present assessments of risk of bias due to missing results (arising from reporting biases) for each synthesis assessed.	Non-Peer-Reviewed and Unpublished Cases; Discussion (known case burden exceeds published cases).
Certainty of evidence	22	Present assessments of certainty (or confidence) in the body of evidence for each outcome assessed.	Discussion (interpreted in light of the limited, low-level evidence base).
**DISCUSSION**			
Discussion	23a	Provide a general interpretation of the results in the context of other evidence.	Discussion.
	23b	Discuss any limitations of the evidence included in the review.	Discussion.
	23c	Discuss any limitations of the review processes used.	Discussion.
	23d	Discuss implications of the results for practice, policy, and future research.	Discussion; Conclusions.
**OTHER INFORMATION**			
Registration and protocol	24a	Provide registration information for the review, including register name and registration number, or state that the review was not registered.	Methods. The review was not registered in a prospective registry (e.g. PROSPERO).
	24b	Indicate where the review protocol can be accessed, or state that a protocol was not prepared.	Methods. No separate protocol was prepared/published; methods are reported in full in the Methods section.
	24c	Describe and explain any amendments to information provided at registration or in the protocol.	Not applicable — no registration or protocol.
Support	25	Describe sources of financial or non-financial support for the review, and the role of the funders or sponsors in the review.	Declarations — Funding (HADDTS Foundation; no role in design, analysis,

Section and Topic	Item #	Checklist item	Location where item is reported
			or reporting).
Competing interests	26	Declare any competing interests of review authors.	Declarations — Competing interests.
Availability of data, code and other materials	27	Report which of the following are publicly available and where they can be found: template data collection forms; data extracted from included studies; data used for all analyses; analytic code; any other materials used in the review.	Declarations — Availability of data and materials; Tables 1–4 and Supplementary Tables/Documents; data drawn from cited primary publications and public databases (PubMed, ClinVar, OMIM, gnomAD).

From: Page MJ, McKenzie JE, Bossuyt PM, Boutron I, Hoffmann TC, Mulrow CD, et al. The PRISMA 2020 statement: an updated guideline for reporting systematic reviews. BMJ 2021;372:n71. doi: 10.1136/bmj.n71.
This work is licensed under CC BY 4.0. To view a copy of this license, visit https://creativecommons.org/licenses/by/4.0/
