# Supplementary material for: Delineating the CTBP1-Related Phenotypic Spectrum: A Review of HADDTS and Atypical Variants
Source: Int J Mol Sci. 2026 Aug 6;27(15):7065. doi: 10.3390/ijms27157065 (PMC13467200; doi:10.3390/ijms27157065)
Supplement: Supplementary file 1 [file ijms-27-07065-s001.zip › Supplementary Document S2..pdf]

Supplementary Document S2.

Supplementary document S2. Official statement from the HADDTS Foundation detailing current known case numbers and registry participation statistics for HADDTS (CTBP1-Related Syndrome) as of May 24, 2026

HADDTS FOUNDATION

Nuremberg, 24 May 2026

On Case Numbers of HADDTS (CTBP1-Related Syndrome) (all CTBP1 mutations):

As of 24 May 2026, the HADDTS Foundation (HF) is aware, through personal communication, of 50 reported cases of individuals carrying a CTBP1 mutation. Of these 50 individuals, 4 died within the past four years, all before reaching the age of 19.

20 families have registered directly with the HF through their website (HADDTS Patient Registry), of whom 9 have provided genetic reports that have been verified by the HADDTS Foundation.

As the HF is funding a Natural History Study and Simons Searchlight already had a database, the HADDTS Foundation has directed its efforts toward increasing case registration within Simons Searchlight.

To date, 25 individuals are registered in Simons Searchlight.

> of which are in status "Consented": 25
> of which are in status "lab report approved": 24
> of which are in status "Completed 1 or more surveys": 19
> of which are in status "Medical History completed": 17

as detailed in https://insight.tempusresearch.com/report/searchlight-participation-progress Participated Gene: "CTBP1"

Although the exact degree of overlap between the 25 cases registered in Simons Searchlight and the 20 families registered directly with the HADDTS Foundation is not known, the two cohorts appear to be closely aligned, as many families are known to participate in both registries. Nevertheless, it cannot be excluded that a small number of cases in either group are not represented in the other, or even among the approximately 50 cases currently known to the HADDTS Foundation. Interest in the condition has grown significantly over the last two years, together with the number of identified cases, following the establishment of the HADDTS Foundation and the preceding work of its founding members.

HADDTS Foundation gGmbH

Benjamin Wüst
Geschäftsführung

Aurelia Contesso
Geschäftsführung
