# Supplementary material for: Delineating the CTBP1-Related Phenotypic Spectrum: A Review of HADDTS and Atypical Variants
Source: Int J Mol Sci. 2026 Aug 6;27(15):7065. doi: 10.3390/ijms27157065 (PMC13467200; doi:10.3390/ijms27157065)
Supplement: Supplementary file 1 [file ijms-27-07065-s001.zip › Supplementary Tables-Revised.pdf]

Supplementary Table S1.

Supplementary Table S1. Loss-of-function variants in *CTBP1* from gnomAD v4.1.1. Variant consequences include frameshift, stop gained, splice acceptor, and splice donor variants as of July 2026. AC = allele count; Hom = homozygote count; ClinVar = ClinVar germline classification; Source = gnomAD dataset (Exomes, Genomes, or Both). gnomAD ID entries are abbreviated for display; long alleles are truncated with '..' to indicate additional sequence. Dash (–) indicates data not available. Source: gnomAD v4.1.1 (*CTBP1*, ENSG00000159692).

nomAD ID	*rsID*	*Protein Change*	*Transcript Change*	*Consequence*	*AC*	*Hom*	*ClinVar*	*Source*
4-1237113-CCGAG-C	rs1188415142	p.Leu66ValfsTer?	c.196_199del	Frameshift	486	7	-	Genomes
4-1237221-AGTGTCCA..-A	rs1731619782	-	c.49-27_91del	Splice acceptor	477	7	Benign	Genomes
4-1237106-G-GGAGAAACC	rs1479839780	p.Ter70ValfsTer?	c.199_206dup	Frameshift	208	5	-	Genomes
4-1237109-GAAACCGA..-G	rs1731602279	p.Tyr45SerfsTer?	c.134_203del	Frameshift	25	1	-	Genomes
4-1212275-C-A	rs868723811	p.Glu419Ter	c.1255G>T	Stop gained	1	0	-	Exomes
4-1212312-CG-C	-	p.Pro406ArgfsTer96	c.1217del	Frameshift	1	0	-	Exomes
4-1212312-C-CGGGGGGG	-	p.His408ProfsTer17	c.1217_1218insCCCCCCC	Frameshift	1	0	-	Exomes
4-1212319-G-GGGGGGGC	-	p.Ala404GlyfsTer21	c.1210_1211insGCCCCCC	Frameshift	1	0	-	Exomes
4-1212324-AG-A	-	p.Pro402LeufsTer100	c.1205del	Frameshift	2	0	-	Exomes
4-1212337-TG-T	-	p.His398ThrfsTer104	c.1192del	Frameshift	1	0	-	Exomes
4-1212356-TG-T	-	p.Ser392AlafsTer110	c.1173del	Frameshift	2	0	-	Exomes
4-1212402-GC-G	-	p.Gly376AlafsTer126	c.1127del	Frameshift	1	0	-	Exomes
4-1212420-G-T	-	p.Tyr370Ter	c.1110C>A	Stop gained	3	0	-	Exomes
4-1212428-T-C	-	-	c.367-2A>G	Splice acceptor	2	0	-	Exomes
4-1213014-GCTGT-G	rs746225680	p.Asp334AlafsTer2	c.1001_1004del	Frameshift	1	0	-	Exomes
4-1213517-G-A	rs1394054108	p.Arg317Ter	c.949C>T	Stop gained	1	0	-	Exomes
4-1213555-T-TG	-	p.His304ProfsTer59	c.910dup	Frameshift	1	0	-	Exomes
4-1213606-C-T	-	-	c.861-1G>A	Splice acceptor	1	0	Uncertain significance	Exomes
4-1214359-C-A	-	p.Glu282Ter	c.844G>T	Stop gained	1	0	-	Exomes
4-1214422-C-A	-	p.Glu261Ter	c.781G>T	Stop gained	1	0	-	Exomes
4-1214435-AC-A	-	p.Gly256ValfsTer13	c.767del	Frameshift	1	0	-	Exomes
4-1223483-C-CA	-	p.Val182CysfsTer16	c.543dup	Frameshift	1	0	-	Genomes
4-1223514-T-C	-	-	c.515-2A>G	Splice acceptor	1	0	-	Exomes
4-1225366-C-A	-	p.Gly170Ter	c.508G>T	Stop gained	1	0	-	Exomes
4-1225373-GC-G	-	p.Gly167AlafsTer56	c.500del	Frameshift	1	0	-	Exomes
4-1225390-G-GTCACCTC..	-	p.Ile161_Arg162insGlyValAlaArgTer	c.483_484insGGGGTGGCGAGGTGA	Stop gained	1	0	-	Genomes
4-1225405-CG-C	-	p.Gly157AlafsTer66	c.468del	Frameshift	1	0	-	Exomes
4-1225444-G-A	rs1425210992	p.Arg144Ter	c.430C>T	Stop gained	1	0	Uncertain significance	Exomes
4-1225487-G-C	rs1388969883	p.Tyr129Ter	c.387C>G	Stop gained	1	0	-	Genomes
4-1228344-CT-C	rs1460287024	-	c.163-2del	Splice acceptor	1	0	-	Genomes
4-1237106-GGAGAAACC-G	rs1479839780	p.Gly67LeufsTer?	c.199_206del	Frameshift	2	0	-	Genomes
4-1237144-CAAACCGA..-C	rs1560271628	p.Tyr45CysfsTer?	c.134_168del	Frameshift	9	0	-	Genomes

4-1237162-C-A	-	p.Glu51Ter	c.151G>T	Stop gained	2	0	-	Exomes
4-1237165-G-A	rs943109492	p.Gln50Ter	c.148C>T	Stop gained	1	0	-	Genomes
4-1237178-ATAAACCG..-A	rs1731613365	p.Pro34SerfsTer2	c.100_134del	Frameshift	4	0	-	Genomes
4-1237179-TAAACCGA..-T	rs1731613606	p.Pro34SerfsTer2	c.99_133del	Frameshift	4	0	-	Genomes
4-1237186-AGTGTCCA..-A	rs1731615619	-	c.49-27_126del	Splice acceptor	7	0	-	Genomes
4-1237208-A-T	rs1325198322	p.Cys35Ter	c.105T>A	Stop gained	742	0	-	Genomes
4-1237213-GCAAACCG..-G	rs1731618417	p.Val22AlafsTer14	c.65_99del	Frameshift	2	0	-	Genomes
4-1237221-AGTGTCCA..-A	rs1731620017	-	c.48+79_91del	Splice acceptor	4	0	-	Genomes
4-1237235-GATGGGGC..-G	rs1731621408	-	c.49-6_77del	Splice acceptor	2	0	-	Both
4-1237247-GA-G	-	p.Val22AlafsTer2	c.65del	Frameshift	2	0	-	Exomes
4-1237248-AC-A	-	p.Val22SerfsTer2	c.64del	Frameshift	5	0	-	Exomes
4-1237256-AGTGTCCA..-A	rs1731624929	-	c.49-27_56del	Splice acceptor	6	0	-	Genomes
4-1237265-C-T	rs1173217980	-	c.49-1G>A	Splice acceptor	3	0	-	Both
4-1237265-C-A	rs1173217980	-	c.49-1G>T	Splice acceptor	1	0	-	Genomes
4-1237326-AGTGTCCA..-A	rs1731636425	-	-	Splice donor	1	0	-	Genomes
4-1237457-TGAAAACC..-T	rs1731654237	-	-	Splice donor	6	0	-	Genomes
4-1237474-C-T	rs1243211172	-	c.48+1G>A	Splice donor	6	0	-	Both
4-1237494-A-AAAACCCC..	rs1731659179	p.Phe10CysfsTer61	c.28_29insGCCCTGAGCCCCATCAGGAGGTGGACACGGGGTTTGTCCTGAGCCCCATCAGGAGGTGGACACGGGGTTT	Frameshift	1	0	-	Genomes
4-1237494-A-AAAACCCC..	rs1731659179	p.Phe10CysfsTer61	c.28_29insGCCCTGTGCCCCATCAGGAGGTGGACACTCGGTTTCTCCTGAGCCCCATCAGGAGGTGGACACGGGGTTT	Frameshift	4	0	-	Genomes
4-1237852-GTGTCCAC..-G	rs1457259493	p.Ile153ThrfsTer52	c.458_492del	Frameshift	90	0	Likely benign	Genomes
4-1237856-C-T	rs1241997369	p.Trp163Ter	c.489G>A	Stop gained	10	0	-	Both
4-1237871-G-T	rs1321037409	p.Cys158Ter	c.474C>A	Stop gained	2	0	-	Both
4-1237873-A-ACAGGGCA..	rs1731724714	p.Cys158SerfsTer47	c.437_471dup	Frameshift	1	0	-	Genomes
4-1237878-GCAAA-G	rs1731725665	p.Phe155ProfsTer37	c.463_466del	Frameshift	9	0	-	Genomes
4-1237878-GCAAACCG..-G	-	p.Glu146ValfsTer59	c.432_466del	Frameshift	1	0	-	Genomes
4-1237882-A-ATTCTT	rs1731725879	p.Phe155LysfsTer40	c.462_463insAAGAA	Frameshift	4	0	-	Genomes
4-1237887-ATGTCCAC..-A	rs1731728932	p.Arg130PhefsTer40	c.388_457del	Frameshift	29	0	-	Genomes
4-1237887-A-ATGTCCAC..	rs755747889	p.Ile153ThrfsTer52	c.423_457dup	Frameshift	123	0	-	Genomes
4-1237900-G-A	rs866710746	p.Gln149Ter	c.445C>T	Stop gained	2	0	-	Exomes
4-1237921-G-GGTGTCCA..	rs777549568	p.His142GlyfsTer63	c.389_423dup	Frameshift	2	0	-	Genomes
4-1237921-GGTGTCCA..-G	rs777549568	p.Arg130ProfsTer5	c.389_423del	Frameshift	7	0	-	Both

4-1237943-ACAGGGAA..-A	rs1731738274	p.Gly111ValfsTer59	c.332_401del	Frameshift	29	0	-	Genomes
4-1237943-A-T	rs1364381689	p.Cys134Ter	c.402T>A	Stop gained	33	0	-	Genomes
4-1237943-A-ACAGGGAA..	-	p.Cys134TrpfsTer36	c.332_401dup	Frameshift	1	0	-	Exomes
4-1237982-GGCAA-G	rs756953087	p.Phe120SerfsTer2	c.359_362del	Frameshift	17	0	-	Exomes
4-1237992-ATGTCCAC..-A	rs1405676396	p.Gly107ArgfsTer28	c.318_352del	Frameshift	25	0	-	Genomes
4-1238005-G-A	rs1292480007	p.Gln114Ter	c.340C>T	Stop gained	3	0	-	Both
4-1238018-G-GC	-	p.Phe109LeufsTer38	c.326_327insG	Frameshift	1	0	-	Genomes
4-1238022-A-AC	-	p.Val108GlyfsTer39	c.322dup	Frameshift	2	0	-	Exomes
4-1238040-G-T	-	p.Ser102Ter	c.305C>A	Stop gained	1	0	-	Exomes
4-1238061-CAG-C	rs747402058	p.Cys95SerfsTer51	c.282_283del	Frameshift	2	0	-	Exomes
4-1238078-C-T	-	p.Trp89Ter	c.267G>A	Stop gained	1	0	-	Exomes
4-1238096-GCT-G	rs769037622	p.Ser83GlnfsTer63	c.247_248del	Frameshift	3	0	-	Exomes
4-1238112-GCCGTGCTC-G	rs1441868905	p.Ser76ArgfsTer68	c.225_232del	Frameshift	1	0	-	Genomes
4-1238134-T-TC	rs781706364	p.Ser71GlufsTer76	c.210dup	Frameshift	2	0	-	Both
4-1238140-C-CA	rs748203299	p.Gly69TrpfsTer78	c.204dup	Frameshift	14	0	-	Both
4-1238209-G-A	-	p.Gln46Ter	c.136C>T	Stop gained	1	0	-	Exomes
4-1238332-G-A	-	p.Arg5Ter	c.13C>T	Stop gained	4	0	-	Exomes
4-1238339-T-C	-	-	c.8-2A>G	Splice acceptor	1	0	-	Exomes
4-1248950-GC-G	-	p.Gly2AlafsTer19	c.5del	Frameshift	1	0	-	Exomes
4-1249665-G-T	-	p.Cys2Ter	c.6C>A	Stop gained	1	0	-	Exomes

Supplementary Table S2.

Supplementary Table S2. External variant-database records (ClinVar and DECIPHER). ClinVar Submissions for *CTBP1* c.991C>T p.Arg331Trp / c.1024C>T p.Arg342Trp from Institutions Without Corresponding Peer-Reviewed Publications (as of July 2026). NR = not reported. Submissions from institutions with corresponding published papers (e.g., Beck et al. (1, 2), Sommerville et al. (3)) are excluded.

Source	Accession / Patient ID	Submitting Institution	Date	Region	Details
**ClinVar**	SCV002548914	New York Genome Center (CSER-NYCKidSeq)	Aug 2021	USA	Hypotonia, ataxia, developmental delay, and tooth enamel defect syndrome; submitted as c.991C>T p.(Arg331Trp), heterozygous; Pathogenic; review status: criteria provided, single submitter (NYGC Assertion Criteria 2020); last evaluated 06 Aug 2021; 1 individual; clinical testing; allele origin de novo; affected status yes; sex, age and clinical features NR.
**ClinVar**	SCV002019759	Revvity Omics (PerkinElmer)	Jan 2019	USA	Hypotonia, ataxia, developmental delay, and tooth enamel defect syndrome; Pathogenic; review status: criteria provided, single submitter (ACMG Guidelines, 2015); last evaluated 08 Jan 2019; 1 observation; clinical testing; allele origin germline; affected status unknown; number of individuals NR; submitted variant nomenclature, interpretive comment and clinical features NR.
**ClinVar**	SCV004801554	Illumina Laboratory Services	Oct 2020	USA	Hypotonia, ataxia, developmental delay, and tooth enamel defect syndrome; submitted as c.1024C>T p.(Arg342Trp), also given as c.991C>T p.(Arg331Trp); Pathogenic; review status: criteria provided, single submitter (ICSL Variant Classification Criteria RUGD, April 2020); last evaluated 27 Oct 2020; 1 observation; clinical testing; allele origin unknown; affected

					status unknown; sex, age and clinical features NR.
**ClinVar**	SCV004933972	Institute of Human Genetics, FAU Erlangen-Nürnberg	Apr 2024	Germany	Hypotonia, ataxia, developmental delay, and tooth enamel defect syndrome (autosomal dominant inheritance); Pathogenic; review status: criteria provided, single submitter (Hauer et al., Genet Med 2018 criteria); last evaluated 23 Apr 2024; 1 individual; clinical testing; allele origin germline; affected status yes; ACMG criteria applied: PS2, PS3, PM2, PP2, PP3; identified by standard clinical testing; submitted variant nomenclature, sex, age and clinical features NR.
**ClinVar**	*SCV007096548	Victorian Clinical Genetics Services / Murdoch Childrens Research Institute	Mar 2025	Australia	Hypotonia, ataxia, developmental delay, and tooth enamel defect syndrome; submitted as p.(Arg331Trp), heterozygous; Pathogenic; review status: criteria provided, single submitter (ACMG Guidelines, 2015); last evaluated 03 Mar 2025; 1 observation; clinical testing; allele origin germline; affected status yes; variant lies in the annotated 2-Hacid_dh domain; submitter states the variant was shown to be paternally inherited by trio analysis; sex, age and clinical features NR; may overlap with Wong et al. (4)
**ClinVar**	SCV002058757	3billion Inc.	Jan 2026	International	Hypotonia, ataxia, developmental delay, and tooth enamel defect syndrome; Pathogenic; review status: criteria provided, single submitter (ACMG Guidelines, 2015); last evaluated 26 Jan 2026; 1 observation; clinical testing, exome sequencing; allele origin germline; affected status yes; absent from gnomAD v4.1.0; REVEL 0.74; number of individuals NR; submitted variant

					nomenclature, sex, age and clinical features NR.
**ClinVar**	SCV007539402	Broad Institute / GREGoR Consortium	Mar 2026	USA	Hypotonia, ataxia, developmental delay, and tooth enamel defect syndrome (autosomal dominant inheritance); submitted as p.Arg331Trp; Pathogenic; review status: criteria provided, single submitter (ACMG Guidelines, 2015); last evaluated 05 Mar 2026; 1 observation; research; allele origin germline; affected status unknown; ACMG criteria applied: PS2_Very Strong, PS3_Supporting, PM2_Supporting, PP3; number of individuals NR; sex, age and clinical features NR.
**DECIPHER**	263727	Not shown in open-access record	NR	Not shown in open-access record	c.1024C>T; p.(Arg342Trp) (ENST00000290921.10); heterozygous; de novo (parentage confirmed); Pathogenic, full contribution (PM2, PS1, PS2, PP3); female, 13 y; aplasia/hypoplasia of the cerebellum, developmental regression, hyporeflexia, lower-limb spasticity, lower-limb joint contracture, thoracolumbar scoliosis, increased CSF protein
**DECIPHER**	303970	Not shown in open-access record	NR	Not shown in open-access record	c.1024C>T; p.(Arg342Trp) (ENST00000290921.10); heterozygous; inheritance unknown; Pathogenic, full contribution (PM2, PS1, PM1, PM6, PP4); female, 5 y 11 mo; abnormal cerebellum morphology, ataxia, global developmental delay, generalised hypotonia, myopathy, short stature
**DECIPHER**	428029	Not shown in open-access record	NR	Not shown in open-access record	c.991C>T; p.(Arg331Trp) (NM_001012614.2 / ENST00000382952.8); heterozygous; inheritance unknown; Pathogenic, full contribution (PM2, PS1, PS3, PP3, PM6); male, 15 y; pontocerebellar atrophy, progressive

	cerebellar ataxia, chronic axonal neuropathy

Note: * submission may correspond to the Australian patient described by Wong et al. (2022) (4).

Supplementary Table S3.

Supplementary Table S3. Copy-number variants overlapping *CTBP1* in DECIPHER. All copy-number variants overlapping *CTBP1* held in DECIPHER v11.40 as of July 2026 are listed (n = 448; 390 deletions and 58 duplications), each reported in a separate patient. Location and Size describe the imbalance on GRCh38. Inheritance, Genotype and Pathogenicity / Contribution are as recorded by the submitting centre; a dash (–) indicates that no classification was submitted. Source: DECIPHER v11.40 (*CTBP1* patient-overlap dataset, ENSG00000159692).

Patient ID	***Location (GRCh38)***	***Size***	***Type***	***Inheritance***	***Genotype***	***Pathogenicity / Contribution***
610	4:65,736–8,260,850	8.20 Mb	Deletion	Unknown	Heterozygous	–
307768	4:71,660–18,838,025	18.77 Mb	Deletion	De novo	Heterozygous	Pathogenic / Full
249910	4:10,001–3,768,544	3.76 Mb	Deletion	De novo	Heterozygous	–
1928	4:206,841–3,305,911	3.10 Mb	Deletion	De novo	Heterozygous	–
4676	4:72,555–3,518,200	3.45 Mb	Deletion	De novo	Heterozygous	–
252491	4:72,355–3,768,685	3.70 Mb	Deletion	Unknown	Heterozygous	–
252660	4:23,423–9,477,538	9.45 Mb	Duplication	Unknown	Heterozygous	–
252667	4:23,423–4,983,510	4.96 Mb	Deletion	Unknown	Heterozygous	–
252766	4:72,773–11,594,528	11.52 Mb	Deletion	Unknown	Heterozygous	–
252785	4:23,423–37,951,193	37.93 Mb	Deletion	Unknown	Heterozygous	–
252884	4:738,223–2,749,033	2.01 Mb	Deletion	Unknown	Heterozygous	–
252978	4:23,423–2,481,190	2.46 Mb	Deletion	Unknown	Heterozygous	–
253029	4:443,891–9,477,538	9.03 Mb	Deletion	Unknown	Heterozygous	–
254484	4:687,750–9,823,154	9.14 Mb	Duplication	Unknown	Heterozygous	–
256909	4:29,099–9,732,961	9.70 Mb	Duplication	De novo	Heterozygous	–
257398	4:68,916–2,033,959	1.97 Mb	Deletion	Unknown	Heterozygous	–
258847	4:75,783–9,374,832	9.30 Mb	Duplication	Imbalance from balanced rearrangement	Heterozygous	–
261438	4:71,660–1,598,712	1.53 Mb	Deletion	De novo	Heterozygous	–
326600	4:72,555–11,173,631	11.10 Mb	Deletion	De novo	Heterozygous	Pathogenic / Full
270953	4:71,660–41,014,251	40.94 Mb	Duplication	Unknown	Heterozygous	–
285503	4:71,660–3,843,370	3.77 Mb	Deletion	Unknown	Heterozygous	Pathogenic
285824	4:71,660–11,932,483	11.86 Mb	Deletion	De novo	Heterozygous	Pathogenic
290287	4:37,335–2,168,956	2.13 Mb	Deletion	De novo	Heterozygous	Pathogenic

327581	4:64,685–10,366,772	10.30 Mb	Deletion	Unknown	Heterozygous	–
331744	4:68,453–3,921,369	3.85 Mb	Deletion	Unknown	Heterozygous	Pathogenic / Full
354618	4:71,660–15,968,877	15.90 Mb	Deletion	Unknown	Heterozygous	Pathogenic
390354	4:10,001–11,322,107	11.31 Mb	Deletion	Imbalance from balanced rearrangement	Heterozygous	Likely pathogenic
391169	4:10,001–11,322,107	11.31 Mb	Deletion	Imbalance from balanced rearrangement	Heterozygous	Likely pathogenic
391172	4:10,001–11,322,107	11.31 Mb	Deletion	Unknown	Heterozygous	Likely pathogenic
392026	4:10,001–11,322,107	11.31 Mb	Deletion	De novo	Heterozygous	Likely pathogenic
392040	4:10,001–11,322,107	11.31 Mb	Deletion	Imbalance from balanced rearrangement	Heterozygous	Likely pathogenic
392066	4:10,002–4,480,201	4.47 Mb	Deletion	De novo	Heterozygous	Likely pathogenic
392072	4:10,002–4,480,201	4.47 Mb	Deletion	De novo	Heterozygous	Likely pathogenic
392082	4:10,001–11,322,107	11.31 Mb	Deletion	De novo	Heterozygous	Likely pathogenic
392265	4:10,001–11,322,107	11.31 Mb	Deletion	Imbalance from balanced rearrangement	Heterozygous	Likely pathogenic
392310	4:10,001–11,322,107	11.31 Mb	Duplication	Imbalance from balanced rearrangement	Heterozygous	Likely pathogenic
393307	4:10,001–11,322,107	11.31 Mb	Deletion	Imbalance from balanced rearrangement	Heterozygous	Likely pathogenic
393324	4:10,001–11,322,107	11.31 Mb	Deletion	Imbalance from balanced rearrangement	Heterozygous	Likely pathogenic
394096	4:10,001–11,322,107	11.31 Mb	Deletion	Imbalance from balanced rearrangement	Heterozygous	Likely pathogenic
394287	4:10,001–11,322,107	11.31 Mb	Deletion	De novo	Heterozygous	Likely pathogenic
394302	4:10,001–5,780,201	5.77 Mb	Deletion	De novo (mosaic)	Heterozygous	Likely pathogenic
395096	4:10,002–4,480,201	4.47 Mb	Deletion	Imbalance from balanced rearrangement	Heterozygous	Likely pathogenic
395300	4:10,001–19,022,108	19.01 Mb	Deletion	Imbalance from balanced rearrangement	Heterozygous	Likely pathogenic
395379	4:10,001–23,122,108	23.11 Mb	Deletion	De novo	Heterozygous	Likely pathogenic
395526	4:10,001–11,322,107	11.31 Mb	Deletion	Imbalance from balanced rearrangement	Heterozygous	Likely pathogenic
395539	4:10,001–4,380,201	4.37 Mb	Deletion	De novo	Heterozygous	Likely pathogenic
395550	4:10,001–11,322,107	11.31 Mb	Deletion	De novo	Heterozygous	Likely pathogenic
395935	4:10,001–19,022,108	19.01 Mb	Deletion	Imbalance from balanced rearrangement	Heterozygous	Likely pathogenic
395941	4:10,001–11,322,107	11.31 Mb	Deletion	De novo	Heterozygous	Likely pathogenic
396062	4:10,001–4,380,201	4.37 Mb	Deletion	Imbalance from balanced rearrangement	Heterozygous	Likely pathogenic
396130	4:10,001–4,380,201	4.37 Mb	Deletion	Imbalance from balanced rearrangement	Heterozygous	Likely pathogenic
396191	4:10,001–19,022,108	19.01 Mb	Deletion	Imbalance from balanced rearrangement	Heterozygous	Likely pathogenic
396334	4:10,001–4,380,201	4.37 Mb	Deletion	Unknown	Heterozygous	Likely pathogenic
396352	4:10,001–11,322,107	11.31 Mb	Deletion	De novo	Heterozygous	Likely pathogenic
396359	4:10,001–11,322,107	11.31 Mb	Deletion	De novo	Heterozygous	Likely pathogenic
396410	4:10,001–4,380,201	4.37 Mb	Deletion	De novo (mosaic)	Heterozygous	Likely pathogenic
396536	4:10,001–19,022,108	19.01 Mb	Deletion	Unknown	Heterozygous	Likely pathogenic
397212	4:10,001–5,780,201	5.77 Mb	Deletion	Imbalance from balanced rearrangement	Heterozygous	Likely pathogenic

397354	4:10,001–11,322,107	11.31 Mb	Duplication	De novo	Heterozygous	Likely pathogenic
398022	4:10,001–4,380,201	4.37 Mb	Deletion	Imbalance from balanced rearrangement	Heterozygous	Likely pathogenic
398394	4:10,001–4,380,201	4.37 Mb	Deletion	Unknown	Heterozygous	Likely pathogenic
400864	4:10,001–11,322,107	11.31 Mb	Deletion	Unknown	Heterozygous	Likely pathogenic
400875	4:10,002–4,480,201	4.47 Mb	Deletion	Unknown	Heterozygous	Likely pathogenic
402410	4:72,355–9,369,490	9.30 Mb	Duplication	De novo	Heterozygous	Likely pathogenic
408231	4:71,660–3,757,341	3.69 Mb	Deletion	De novo	Heterozygous	Pathogenic / Partial
421080	4:71,660–3,792,983	3.72 Mb	Deletion	Unknown	Heterozygous	Pathogenic
427255	4:118,051–2,515,720	2.40 Mb	Deletion	De novo	Heterozygous	–
428975	4:394,555–3,870,653	3.48 Mb	Deletion	De novo	Heterozygous	Pathogenic / Full
430250	4:72,555–2,231,949	2.16 Mb	Deletion	Unknown	Heterozygous	Pathogenic
452816	4:72,555–9,369,341	9.30 Mb	Deletion	Unknown	Heterozygous	Pathogenic
456527	4:1,231,023–2,322,109	1.09 Mb	Deletion	Unknown	Heterozygous	Pathogenic / Full
472214	4:49,556–1,874,520	1.82 Mb	Deletion	Unknown	Heterozygous	Pathogenic
472387	4:49,556–5,514,775	5.47 Mb	Deletion	Unknown	Heterozygous	Pathogenic
487040	4:71,660–3,142,853	3.07 Mb	Deletion	Unknown	Heterozygous	Pathogenic
503370	4:71,660–6,180,173	6.11 Mb	Deletion	De novo	Heterozygous	Pathogenic / Full
508833	4:68,454–22,047,113	21.98 Mb	Duplication	Imbalance from balanced rearrangement	Heterozygous	–
530483	4:1–3,820,606	3.82 Mb	Deletion	Unknown	Heterozygous	Pathogenic
534691	4:72,555–2,325,477	2.25 Mb	Deletion	Unknown	Heterozygous	–
534983	4:71,660–14,576,531	14.50 Mb	Deletion	Unknown	Heterozygous	Pathogenic / Full
535066	4:72,555–7,896,242	7.82 Mb	Deletion	Unknown	Heterozygous	Pathogenic / Full
564571	4:49,556–15,352,265	15.30 Mb	Deletion	De novo (confirmed)	Heterozygous	Pathogenic
564574	4:49,556–15,098,376	15.05 Mb	Deletion	De novo (confirmed)	Heterozygous	Pathogenic
564580	4:49,556–1,393,992	1.34 Mb	Deletion	De novo (confirmed)	Heterozygous	Pathogenic
564581	4:49,556–6,248,455	6.20 Mb	Deletion	De novo (confirmed)	Heterozygous	Pathogenic
564598	4:49,556–8,871,251	8.82 Mb	Deletion	De novo (confirmed)	Heterozygous	Pathogenic
564599	4:49,556–3,879,305	3.83 Mb	Deletion	De novo (confirmed)	Heterozygous	Pathogenic
564605	4:49,556–5,947,831	5.90 Mb	Deletion	De novo (confirmed)	Heterozygous	Pathogenic
564607	4:49,556–22,026,951	21.98 Mb	Deletion	De novo (confirmed)	Heterozygous	Pathogenic
564608	4:933,992–12,593,514	11.66 Mb	Duplication	De novo (confirmed)	Heterozygous	Likely pathogenic
564609	4:49,556–17,635,043	17.59 Mb	Deletion	De novo (confirmed)	Heterozygous	Pathogenic
564614	4:49,556–7,209,470	7.16 Mb	Deletion	De novo (confirmed)	Heterozygous	Pathogenic
564622	4:49,556–6,415,301	6.37 Mb	Deletion	De novo (confirmed)	Heterozygous	Pathogenic
564629	4:70,489–4,444,148	4.37 Mb	Deletion	De novo (confirmed)	Heterozygous	Pathogenic

564640	4:49,556–8,918,489	8.87 Mb	Deletion	Imbalance from balanced rearrangement	Heterozygous	Pathogenic
564641	4:49,556–3,024,660	2.98 Mb	Deletion	De novo (confirmed)	Heterozygous	Pathogenic
564650	4:49,556–6,194,694	6.15 Mb	Deletion	De novo (confirmed)	Heterozygous	Pathogenic
564651	4:49,556–2,894,698	2.85 Mb	Deletion	De novo (confirmed)	Heterozygous	Pathogenic
564662	4:49,556–15,077,909	15.03 Mb	Deletion	De novo (confirmed)	Heterozygous	Pathogenic
564665	4:49,556–16,799,418	16.75 Mb	Deletion	De novo (confirmed)	Heterozygous	Pathogenic
564670	4:49,556–9,775,640	9.73 Mb	Deletion	De novo (confirmed)	Heterozygous	Pathogenic
564671	4:49,556–8,867,934	8.82 Mb	Deletion	De novo (confirmed)	Heterozygous	Pathogenic
564678	4:49,556–3,502,303	3.45 Mb	Deletion	De novo (confirmed)	Heterozygous	Pathogenic
564680	4:49,556–5,947,831	5.90 Mb	Deletion	De novo (confirmed)	Heterozygous	Pathogenic
564684	4:49,556–10,001,187	9.95 Mb	Deletion	De novo (confirmed)	Heterozygous	Pathogenic
564686	4:49,556–9,775,640	9.73 Mb	Deletion	De novo (confirmed)	Heterozygous	Pathogenic
564688	4:49,556–14,007,382	13.96 Mb	Deletion	De novo (confirmed)	Heterozygous	Pathogenic
564694	4:49,556–5,768,548	5.72 Mb	Deletion	De novo (confirmed)	Heterozygous	Pathogenic
569355	4:72,555–20,562,383	20.49 Mb	Deletion	Unknown	Heterozygous	Pathogenic / Uncertain
306997	4:71,660–8,279,391	8.21 Mb	Deletion	Unknown	Heterozygous	Likely pathogenic
253146	4:72,555–3,768,544	3.70 Mb	Deletion	De novo	Heterozygous	–
295524	4:933,992–12,593,514	11.66 Mb	Duplication	Unknown	Heterozygous	Pathogenic
259061	4:68,267–13,261,412	13.19 Mb	Deletion	De novo	Heterozygous	–
267148	4:71,660–8,172,198	8.10 Mb	Deletion	Unknown	Heterozygous	–
2616	4:51,519–3,817,215	3.77 Mb	Deletion	Unknown	Heterozygous	–
2841	4:1,081,829–8,098,836	7.02 Mb	Duplication	Unknown	Heterozygous	–
252479	4:72,355–6,123,291	6.05 Mb	Deletion	Unknown	Heterozygous	–
252482	4:72,355–8,666,025	8.59 Mb	Deletion	Unknown	Heterozygous	–
252627	4:23,423–15,319,558	15.30 Mb	Deletion	Unknown	Heterozygous	–
252631	4:23,423–2,481,190	2.46 Mb	Deletion	Unknown	Heterozygous	–
252663	4:23,423–2,696,412	2.67 Mb	Deletion	Unknown	Heterozygous	–
252722	4:23,423–9,440,675	9.42 Mb	Duplication	Unknown	Heterozygous	–
252804	4:23,423–2,696,412	2.67 Mb	Deletion	Unknown	Heterozygous	–
252851	4:65,736–4,124,745	4.06 Mb	Deletion	Unknown	Heterozygous	–
252868	4:23,423–1,625,662	1.60 Mb	Deletion	Unknown	Heterozygous	–
252871	4:23,423–10,162,804	10.14 Mb	Deletion	Unknown	Heterozygous	–
252947	4:23,423–8,728,947	8.71 Mb	Deletion	Unknown	Heterozygous	–
252974	4:72,773–17,129,069	17.06 Mb	Deletion	Unknown	Heterozygous	–
252992	4:23,423–20,082,025	20.06 Mb	Deletion	Unknown	Heterozygous	–

254927	4:72,555–2,754,330	2.68 Mb	Deletion	Imbalance from balanced rearrangement	Heterozygous	–
255798	4:72,555–18,631,896	18.56 Mb	Deletion	De novo	Heterozygous	–
259621	4:36,925–9,402,386	9.37 Mb	Duplication	De novo	Heterozygous	–
261436	4:71,660–1,409,532	1.34 Mb	Deletion	Inherited (affected parent)	Heterozygous	–
263141	4:71,660–3,775,116	3.70 Mb	Deletion	Unknown	Heterozygous	–
266946	4:477,563–4,188,520	3.71 Mb	Deletion	De novo	Heterozygous	–
269158	4:10,001–15,000,033	14.99 Mb	Duplication	Unknown	Heterozygous	–
271776	4:10,001–3,901,856	3.89 Mb	Deletion	De novo	Heterozygous	–
272155	4:119,190–2,008,000	1.89 Mb	Deletion	De novo	Heterozygous	–
272402	4:114,099–4,937,923	4.82 Mb	Deletion	Unknown	Heterozygous	–
279894	4:75,784–1,818,268	1.74 Mb	Deletion	Unknown	Heterozygous	–
280834	4:72,555–8,665,325	8.59 Mb	Deletion	De novo	Heterozygous	–
286021	4:62,355–1,376,545	1.31 Mb	Deletion	De novo	Heterozygous	Pathogenic
288001	4:71,661–6,123,164	6.05 Mb	Deletion	De novo	Heterozygous	Pathogenic
288736	4:1,246,792–1,313,288	66 kb	Duplication	Maternal	Heterozygous	Uncertain significance
327134	4:116,762–2,615,683	2.50 Mb	Duplication	Unknown	Heterozygous	Pathogenic
341526	4:71,660–2,092,689	2.02 Mb	Deletion	Unknown	Heterozygous	Pathogenic / Full
361089	4:10,001–1,727,768	1.72 Mb	Deletion	De novo	Heterozygous	Likely pathogenic / Partial
363852	4:71,660–21,043,954	20.97 Mb	Deletion	Unknown	Heterozygous	Pathogenic / Full
366489	4:71,660–3,843,370	3.77 Mb	Deletion	Unknown	Heterozygous	Uncertain significance
381484	4:33,574–3,973,991	3.94 Mb	Duplication	Unknown	Heterozygous	Likely benign / Uncertain
392060	4:10,001–11,322,107	11.31 Mb	Deletion	Imbalance from balanced rearrangement	Heterozygous	Likely pathogenic
392068	4:10,001–11,322,107	11.31 Mb	Deletion	Imbalance from balanced rearrangement	Heterozygous	Likely pathogenic
392073	4:10,001–11,322,107	11.31 Mb	Deletion	De novo	Heterozygous	Likely pathogenic
392250	4:10,001–11,322,107	11.31 Mb	Deletion	De novo	Heterozygous	Likely pathogenic
393919	4:10,001–4,380,201	4.37 Mb	Deletion	De novo	Heterozygous	Likely pathogenic
394812	4:10,001–11,322,107	11.31 Mb	Deletion	Imbalance from balanced rearrangement	Heterozygous	Likely pathogenic
394819	4:10,001–11,322,107	11.31 Mb	Deletion	Imbalance from balanced rearrangement	Heterozygous	Likely pathogenic
395372	4:10,001–19,022,108	19.01 Mb	Duplication	Imbalance from balanced rearrangement	Heterozygous	Likely pathogenic
395502	4:10,001–15,822,108	15.81 Mb	Deletion	De novo	Heterozygous	Likely pathogenic
395515	4:10,001–11,322,107	11.31 Mb	Deletion	De novo	Heterozygous	Likely pathogenic
395537	4:10,001–23,122,108	23.11 Mb	Deletion	Imbalance from balanced rearrangement	Heterozygous	Likely pathogenic
395926	4:10,001–19,022,108	19.01 Mb	Deletion	Imbalance from balanced rearrangement	Heterozygous	Likely pathogenic
395948	4:10,001–4,380,201	4.37 Mb	Deletion	De novo	Heterozygous	Likely pathogenic

395966	4:10,001–11,322,107	11.31 Mb	Deletion	De novo	Heterozygous	Likely pathogenic
396049	4:10,001–4,380,201	4.37 Mb	Deletion	Imbalance from balanced rearrangement	Heterozygous	Likely pathogenic
396132	4:10,001–19,022,108	19.01 Mb	Deletion	De novo	Heterozygous	Likely pathogenic
396171	4:10,001–4,380,201	4.37 Mb	Deletion	Unknown	Heterozygous	Likely pathogenic
396375	4:10,001–4,380,201	4.37 Mb	Deletion	Unknown	Heterozygous	Likely pathogenic
396483	4:10,001–4,380,201	4.37 Mb	Duplication	Imbalance from balanced rearrangement	Heterozygous	Likely pathogenic
396545	4:10,001–4,380,201	4.37 Mb	Deletion	De novo	Heterozygous	Likely pathogenic
397390	4:10,001–4,380,201	4.37 Mb	Deletion	De novo	Heterozygous	Likely pathogenic
398075	4:10,001–4,380,201	4.37 Mb	Deletion	Imbalance from balanced rearrangement	Heterozygous	Likely pathogenic
398099	4:10,001–4,380,201	4.37 Mb	Deletion	Imbalance from balanced rearrangement	Heterozygous	Likely pathogenic
398364	4:10,001–4,380,201	4.37 Mb	Deletion	Imbalance from balanced rearrangement	Heterozygous	Likely pathogenic
399086	4:10,001–4,380,201	4.37 Mb	Deletion	Imbalance from balanced rearrangement	Heterozygous	Likely pathogenic
400870	4:10,001–11,322,107	11.31 Mb	Deletion	Unknown	Heterozygous	Likely pathogenic
400883	4:10,001–23,022,108	23.01 Mb	Deletion	Unknown	Heterozygous	Likely pathogenic
401673	4:68,454–1,371,836	1.30 Mb	Deletion	De novo	Heterozygous	Likely pathogenic
414266	4:68,453–8,701,094	8.63 Mb	Deletion	Unknown	Heterozygous	Pathogenic
427535	4:1,068,333–1,536,035	468 kb	Duplication	Unknown	Heterozygous	Uncertain significance / Uncertain
428002	4:71,660–6,479,683	6.41 Mb	Deletion	Unknown	Heterozygous	Likely pathogenic / Uncertain
428003	4:71,660–13,395,123	13.32 Mb	Deletion	Unknown	Heterozygous	Likely pathogenic / Full
472196	4:49,556–16,004,076	15.95 Mb	Deletion	Unknown	Heterozygous	Pathogenic
472596	4:49,556–14,802,793	14.75 Mb	Deletion	Unknown	Heterozygous	Pathogenic
472822	4:49,556–8,569,246	8.52 Mb	Deletion	Unknown	Heterozygous	Pathogenic
472910	4:49,556–8,871,086	8.82 Mb	Deletion	Unknown	Heterozygous	Pathogenic
482857	4:68,454–9,896,433	9.83 Mb	Duplication	Unknown	Heterozygous	Pathogenic / Partial
487184	4:71,660–14,931,238	14.86 Mb	Deletion	Unknown	Heterozygous	Pathogenic
503400	4:71,660–1,409,532	1.34 Mb	Deletion	Imbalance from balanced rearrangement	Heterozygous	Pathogenic / Full
508359	4:49,556–9,442,168	9.39 Mb	Duplication	De novo	Heterozygous	Pathogenic / Partial
515322	4:12,985–10,099,333	10.09 Mb	Duplication	Unknown	Heterozygous	Likely pathogenic / Full
530478	4:1–9,439,769	9.44 Mb	Duplication	Unknown	Heterozygous	Pathogenic
530485	4:1–3,920,606	3.92 Mb	Deletion	Unknown	Heterozygous	Pathogenic
531381	4:71,660–3,768,544	3.70 Mb	Duplication	Unknown	Heterozygous	Likely pathogenic / Partial
534516	4:72,555–3,847,154	3.77 Mb	Deletion	Unknown	Heterozygous	–

564570	4:49,556–8,673,922	8.62 Mb	Deletion	Imbalance from balanced rearrangement	Heterozygous	Pathogenic
564576	4:49,556–22,899,724	22.85 Mb	Deletion	De novo (confirmed)	Heterozygous	Pathogenic
564583	4:49,556–2,008,670	1.96 Mb	Deletion	De novo (confirmed)	Heterozygous	Pathogenic
564588	4:49,556–3,198,133	3.15 Mb	Deletion	Imbalance from balanced rearrangement	Heterozygous	Pathogenic
564592	4:49,556–9,544,312	9.49 Mb	Deletion	Imbalance from balanced rearrangement	Heterozygous	Pathogenic
564606	4:49,556–10,412,284	10.36 Mb	Deletion	De novo (confirmed)	Heterozygous	Pathogenic
564612	4:49,556–1,583,297	1.53 Mb	Deletion	Imbalance from balanced rearrangement	Heterozygous	Pathogenic
564618	4:49,556–2,384,684	2.34 Mb	Deletion	De novo (confirmed)	Heterozygous	Pathogenic
564627	4:49,556–15,465,676	15.42 Mb	Deletion	De novo (confirmed)	Heterozygous	Pathogenic
564637	4:49,556–23,730,587	23.68 Mb	Deletion	De novo (confirmed)	Heterozygous	Pathogenic
564638	4:49,556–7,098,273	7.05 Mb	Deletion	De novo (confirmed)	Heterozygous	Pathogenic
564643	4:49,556–19,904,652	19.86 Mb	Deletion	De novo (confirmed)	Heterozygous	Pathogenic
564645	4:49,556–6,521,293	6.47 Mb	Deletion	De novo (confirmed)	Heterozygous	Pathogenic
564647	4:49,556–3,978,411	3.93 Mb	Deletion	Imbalance from balanced rearrangement	Heterozygous	Pathogenic
564654	4:49,556–8,871,251	8.82 Mb	Deletion	De novo (confirmed)	Heterozygous	Pathogenic
564660	4:49,556–3,764,250	3.71 Mb	Deletion	De novo (confirmed)	Heterozygous	Pathogenic
564663	4:49,556–7,487,137	7.44 Mb	Deletion	De novo (confirmed)	Heterozygous	Pathogenic
564664	4:49,556–12,006,787	11.96 Mb	Deletion	De novo (confirmed)	Heterozygous	Pathogenic
564669	4:49,556–2,193,297	2.14 Mb	Deletion	De novo (confirmed)	Heterozygous	Pathogenic
564676	4:49,556–12,898,612	12.85 Mb	Deletion	De novo (confirmed)	Heterozygous	Pathogenic
564677	4:49,556–11,589,152	11.54 Mb	Deletion	De novo (confirmed)	Heterozygous	Pathogenic
564687	4:49,556–12,001,443	11.95 Mb	Deletion	De novo (confirmed)	Heterozygous	Pathogenic
564691	4:49,556–3,870,653	3.82 Mb	Deletion	De novo (confirmed)	Heterozygous	Pathogenic
564693	4:49,556–2,570,020	2.52 Mb	Deletion	De novo (confirmed)	Heterozygous	Pathogenic
564695	4:49,556–8,918,489	8.87 Mb	Deletion	De novo (confirmed)	Heterozygous	Pathogenic
564697	4:49,556–7,024,977	6.98 Mb	Deletion	De novo (confirmed)	Heterozygous	Pathogenic
564699	4:49,556–11,635,376	11.59 Mb	Deletion	De novo (confirmed)	Heterozygous	Pathogenic
564701	4:49,556–16,498,377	16.45 Mb	Deletion	De novo (confirmed)	Heterozygous	Pathogenic
564703	4:49,556–23,898,377	23.85 Mb	Deletion	De novo (confirmed)	Heterozygous	Pathogenic
564704	4:49,556–1,316,134	1.27 Mb	Deletion	De novo (confirmed)	Heterozygous	Pathogenic
308723	4:72,555–16,552,464	16.48 Mb	Duplication	Unknown	Heterozygous	Pathogenic / Full
281038	4:10,001–7,287,692	7.28 Mb	Duplication	Unknown	Heterozygous	–
296442	4:71,660–38,138,383	38.07 Mb	Duplication	Unknown	Heterozygous	–
288139	4:71,661–9,556,518	9.48 Mb	Duplication	Unknown	Heterozygous	Uncertain significance
275669	4:10,001–8,730,978	8.72 Mb	Deletion	De novo	Heterozygous	–

301250	4:10,001–3,901,856	3.89 Mb	Deletion	Unknown	Heterozygous	Pathogenic / Full
302018	4:149,629–1,873,588	1.72 Mb	Deletion	De novo	Heterozygous	Pathogenic / Full
1893	4:1,081,810–5,238,281	4.16 Mb	Deletion	Unknown	Heterozygous	–
251103	4:72,555–10,140,365	10.07 Mb	Duplication	Unknown	Heterozygous	–
252891	4:23,423–5,679,933	5.66 Mb	Deletion	Unknown	Heterozygous	–
258407	4:72,555–4,409,420	4.34 Mb	Deletion	De novo	Heterozygous	–
259597	4:72,355–10,746,402	10.67 Mb	Duplication	Imbalance from balanced rearrangement	Heterozygous	–
260581	4:68,453–2,244,916	2.18 Mb	Duplication	Imbalance from balanced rearrangement	Heterozygous	–
261474	4:71,660–3,843,370	3.77 Mb	Deletion	Imbalance from balanced rearrangement	Heterozygous	–
267765	4:37,151–14,748,494	14.71 Mb	Duplication	Imbalance from balanced rearrangement	Heterozygous	–
276276	4:56,934–4,188,379	4.13 Mb	Deletion	Unknown	Heterozygous	–
276539	4:10,001–48,189,139	48.18 Mb	Duplication	Unknown	Heterozygous	–
277460	4:71,660–3,870,653	3.80 Mb	Deletion	Unknown	Heterozygous	–
279008	4:1,197,122–2,486,916	1.29 Mb	Duplication	Maternal	Heterozygous	–
284559	4:75,784–7,896,213	7.82 Mb	Deletion	Unknown	Heterozygous	Pathogenic
289569	4:71,661–3,879,183	3.81 Mb	Duplication	De novo	Heterozygous	Pathogenic
289649	4:71,661–1,553,036	1.48 Mb	Deletion	De novo	Heterozygous	Pathogenic
263569	4:71,660–3,373,910	3.30 Mb	Deletion	De novo (confirmed)	Heterozygous	Pathogenic
339704	4:71,660–3,870,653	3.80 Mb	Deletion	De novo	Heterozygous	Pathogenic / Partial
339928	4:71,660–1,798,698	1.73 Mb	Deletion	Unknown	Heterozygous	–
349806	4:33,574–3,792,897	3.76 Mb	Deletion	Unknown	Heterozygous	Likely pathogenic / Partial
392049	4:10,001–11,322,107	11.31 Mb	Deletion	Imbalance from balanced rearrangement	Heterozygous	Likely pathogenic
392055	4:10,001–11,322,107	11.31 Mb	Deletion	Imbalance from balanced rearrangement	Heterozygous	Likely pathogenic
392076	4:10,001–11,322,107	11.31 Mb	Deletion	De novo	Heterozygous	Likely pathogenic
392644	4:10,001–11,322,107	11.31 Mb	Deletion	Imbalance from balanced rearrangement	Heterozygous	Likely pathogenic
392774	4:10,001–11,322,107	11.31 Mb	Deletion	Imbalance from balanced rearrangement	Heterozygous	Likely pathogenic
392790	4:10,001–11,322,107	11.31 Mb	Deletion	Imbalance from balanced rearrangement	Heterozygous	Likely pathogenic
395503	4:10,001–11,322,107	11.31 Mb	Deletion	De novo	Heterozygous	Likely pathogenic
395557	4:10,001–21,298,377	21.29 Mb	Deletion	Imbalance from balanced rearrangement	Heterozygous	Pathogenic / Full
395559	4:10,001–11,322,107	11.31 Mb	Deletion	Imbalance from balanced rearrangement	Heterozygous	Likely pathogenic
396163	4:10,001–4,380,201	4.37 Mb	Deletion	Unknown	Heterozygous	Likely pathogenic
396179	4:10,001–4,380,201	4.37 Mb	Deletion	De novo	Heterozygous	Likely pathogenic
396351	4:10,001–24,898,378	24.89 Mb	Duplication	Imbalance from balanced rearrangement	Heterozygous	Pathogenic / Full
396511	4:10,001–11,322,107	11.31 Mb	Deletion	De novo	Heterozygous	Likely pathogenic

398344	4:10,001–4,380,201	4.37 Mb	Deletion	Unknown	Heterozygous	Likely pathogenic
398406	4:10,001–4,380,201	4.37 Mb	Deletion	Unknown	Heterozygous	Likely pathogenic
398414	4:10,001–4,380,201	4.37 Mb	Deletion	Unknown	Heterozygous	Likely pathogenic
400945	4:891,868–1,719,562	828 kb	Duplication	Unknown	Heterozygous	Likely pathogenic
402205	4:106,802–13,766,267	13.66 Mb	Deletion	Imbalance from balanced rearrangement	Heterozygous	Likely pathogenic
402211	4:10,001–11,322,107	11.31 Mb	Deletion	De novo	Heterozygous	Likely pathogenic
402695	4:10,001–4,380,201	4.37 Mb	Deletion	Imbalance from balanced rearrangement	Heterozygous	Likely pathogenic
402721	4:10,002–4,380,201	4.37 Mb	Deletion	De novo	Heterozygous	Likely pathogenic
411636	4:71,660–15,548,786	15.48 Mb	Duplication	Unknown	Heterozygous	Pathogenic
421071	4:49,556–2,558,263	2.51 Mb	Deletion	De novo	Heterozygous	Pathogenic
428835	4:1,152,978–3,032,629	1.88 Mb	Duplication	Unknown	Heterozygous	Uncertain significance / Uncertain
454777	4:73,108–4,248,962	4.18 Mb	Duplication	Unknown	Heterozygous	Pathogenic / Partial
472495	4:49,556–2,067,163	2.02 Mb	Deletion	Unknown	Heterozygous	Pathogenic
472587	4:49,556–5,095,230	5.05 Mb	Deletion	Unknown	Heterozygous	Pathogenic
472860	4:49,556–1,827,274	1.78 Mb	Deletion	Unknown	Heterozygous	Pathogenic
488435	4:51,519–3,871,253	3.82 Mb	Deletion	Imbalance from balanced rearrangement	Heterozygous	Likely pathogenic / Partial
515374	4:49,556–4,347,478	4.30 Mb	Deletion	Unknown	Heterozygous	Pathogenic / Full
530479	4:1–13,789,606	13.79 Mb	Deletion	Unknown	Heterozygous	Pathogenic
530482	4:1–2,570,606	2.57 Mb	Deletion	Unknown	Heterozygous	Pathogenic
562946	4:49,556–15,098,376	15.05 Mb	Deletion	De novo	Heterozygous	Pathogenic / Full
564569	4:49,556–3,454,273	3.40 Mb	Deletion	De novo (confirmed)	Heterozygous	Pathogenic
564572	4:49,556–5,552,030	5.50 Mb	Deletion	De novo (confirmed)	Heterozygous	Pathogenic
564573	4:49,556–2,400,979	2.35 Mb	Deletion	De novo (confirmed)	Heterozygous	Pathogenic
564577	4:49,556–2,988,772	2.94 Mb	Deletion	De novo (confirmed)	Heterozygous	Pathogenic
564578	4:49,556–2,558,263	2.51 Mb	Deletion	De novo (confirmed)	Heterozygous	Pathogenic
564586	4:49,556–11,699,569	11.65 Mb	Deletion	De novo (confirmed)	Heterozygous	Pathogenic
564586	4:49,556–11,699,569	11.65 Mb	Duplication	De novo (confirmed)	Heterozygous	Pathogenic
564587	4:49,556–14,851,867	14.80 Mb	Deletion	De novo (confirmed)	Heterozygous	Pathogenic
564591	4:49,556–8,988,174	8.94 Mb	Deletion	Imbalance from balanced rearrangement	Heterozygous	Pathogenic
564596	4:49,556–15,813,191	15.76 Mb	Deletion	De novo (confirmed)	Heterozygous	Pathogenic
564601	4:49,556–4,098,232	4.05 Mb	Deletion	De novo (confirmed)	Heterozygous	Pathogenic
564603	4:49,556–11,932,483	11.88 Mb	Deletion	De novo (confirmed)	Heterozygous	Pathogenic
564604	4:49,556–2,008,670	1.96 Mb	Deletion	De novo (confirmed)	Heterozygous	Pathogenic
564615	4:49,556–3,942,257	3.89 Mb	Deletion	De novo (confirmed)	Heterozygous	Pathogenic

564616	4:49,556–4,899,273	4.85 Mb	Deletion	De novo (confirmed)	Heterozygous	Pathogenic
564619	4:69,569–6,621,605	6.55 Mb	Deletion	De novo (confirmed)	Heterozygous	Pathogenic
564625	4:49,556–5,552,407	5.50 Mb	Deletion	De novo (confirmed)	Heterozygous	Pathogenic
564626	4:49,556–9,369,390	9.32 Mb	Deletion	De novo (confirmed)	Heterozygous	Pathogenic
564636	4:49,556–15,658,026	15.61 Mb	Deletion	De novo (confirmed)	Heterozygous	Pathogenic
564639	4:49,556–8,355,918	8.31 Mb	Deletion	De novo (confirmed)	Heterozygous	Pathogenic
564646	4:49,556–13,056,246	13.01 Mb	Deletion	De novo (confirmed)	Heterozygous	Pathogenic
564652	4:49,556–14,233,006	14.18 Mb	Deletion	De novo (confirmed)	Heterozygous	Pathogenic
564653	4:49,556–7,864,773	7.82 Mb	Deletion	De novo (confirmed)	Heterozygous	Pathogenic
564656	4:49,556–11,053,808	11.00 Mb	Deletion	De novo (confirmed)	Heterozygous	Uncertain significance
564657	4:690,053–3,476,596	2.79 Mb	Deletion	De novo (confirmed)	Heterozygous	Pathogenic
564658	4:49,556–7,585,197	7.54 Mb	Deletion	De novo (confirmed)	Heterozygous	Pathogenic
564661	4:49,556–3,765,667	3.72 Mb	Deletion	De novo (confirmed)	Heterozygous	Pathogenic
564667	4:49,556–10,727,135	10.68 Mb	Deletion	De novo (confirmed)	Heterozygous	Pathogenic
564672	4:49,556–2,479,763	2.43 Mb	Deletion	De novo (confirmed)	Heterozygous	Pathogenic
564673	4:49,556–10,856,368	10.81 Mb	Deletion	De novo (confirmed)	Heterozygous	Pathogenic
564674	4:49,556–16,570,608	16.52 Mb	Deletion	De novo (confirmed)	Heterozygous	Pathogenic
564683	4:49,556–8,864,824	8.82 Mb	Deletion	De novo (confirmed)	Heterozygous	Pathogenic
564685	4:49,556–3,918,703	3.87 Mb	Deletion	De novo (confirmed)	Heterozygous	Pathogenic
564689	4:49,556–12,007,438	11.96 Mb	Deletion	De novo (confirmed)	Heterozygous	Pathogenic
564690	4:49,556–4,165,406	4.12 Mb	Deletion	De novo (confirmed)	Heterozygous	Pathogenic
564702	4:49,556–8,636,284	8.59 Mb	Deletion	De novo (confirmed)	Heterozygous	Pathogenic
568958	4:72,555–1,962,072	1.89 Mb	Deletion	Unknown	Heterozygous	Pathogenic / Partial
314306	4:72,428–3,847,029	3.77 Mb	Deletion	Unknown	Heterozygous	Pathogenic
306821	4:71,660–5,811,236	5.74 Mb	Deletion	Unknown	Heterozygous	Uncertain significance
283051	4:10,001–10,004,165	9.99 Mb	Duplication	De novo	Heterozygous	–
293489	4:71,660–5,504,861	5.43 Mb	Deletion	Unknown	Heterozygous	Pathogenic / Full
296428	4:72,555–18,631,896	18.56 Mb	Duplication	Unknown	Heterozygous	–
296432	4:71,660–5,895,278	5.82 Mb	Duplication	Unknown	Heterozygous	Likely pathogenic
262183	4:689,662–1,911,005	1.22 Mb	Deletion	Unknown	Heterozygous	–
288500	4:71,661–1,612,159	1.54 Mb	Deletion	Unknown	Heterozygous	Uncertain significance
287892	4:71,661–8,731,010	8.66 Mb	Deletion	De novo	Heterozygous	Pathogenic
1896	4:51,519–2,077,703	2.03 Mb	Deletion	Unknown	Heterozygous	–
2368	4:65,736–8,260,850	8.20 Mb	Deletion	Unknown	Heterozygous	–
2599	4:1,081,810–3,586,192	2.50 Mb	Deletion	De novo	Heterozygous	–

2797	4:72,555–1,808,363	1.74 Mb	Deletion	Unknown	Heterozygous	–
249481	4:65,736–3,752,027	3.69 Mb	Deletion	Unknown	Heterozygous	–
251921	4:72,753–3,089,017	3.02 Mb	Duplication	Inherited (unaffected parent)	Heterozygous	–
252481	4:72,355–8,721,115	8.65 Mb	Duplication	Unknown	Heterozygous	–
252639	4:65,736–2,628,183	2.56 Mb	Deletion	Unknown	Heterozygous	–
252727	4:72,773–9,604,165	9.53 Mb	Deletion	Unknown	Heterozygous	–
252767	4:23,423–2,350,008	2.33 Mb	Deletion	Unknown	Heterozygous	–
252810	4:72,773–9,477,538	9.40 Mb	Deletion	Unknown	Heterozygous	–
252826	4:23,423–3,621,594	3.60 Mb	Deletion	Unknown	Heterozygous	–
252854	4:23,423–2,119,290	2.10 Mb	Deletion	Unknown	Heterozygous	–
252969	4:443,891–15,319,558	14.88 Mb	Deletion	Unknown	Heterozygous	–
253144	4:72,555–2,500,968	2.43 Mb	Deletion	De novo	Heterozygous	–
254333	4:1,207,123–2,568,089	1.36 Mb	Deletion	Unknown	Heterozygous	–
256755	4:68,267–1,598,653	1.53 Mb	Deletion	De novo	Heterozygous	–
257053	4:162,310–1,692,994	1.53 Mb	Deletion	Imbalance from balanced rearrangement	Heterozygous	–
262054	4:37,135–9,369,399	9.33 Mb	Deletion	De novo	Heterozygous	–
266264	4:72,555–25,819,984	25.75 Mb	Deletion	Unknown	Heterozygous	–
267111	4:45,886–11,805,650	11.76 Mb	Duplication	Imbalance from balanced rearrangement	Heterozygous	–
270318	4:68,453–12,546,292	12.48 Mb	Duplication	Unknown	Heterozygous	–
273371	4:68,453–2,349,163	2.28 Mb	Deletion	Unknown	Heterozygous	–
327610	4:71,528–3,389,173	3.32 Mb	Deletion	Unknown	Heterozygous	–
339326	4:71,660–7,403,689	7.33 Mb	Deletion	Unknown	Heterozygous	–
357833	4:71,660–3,843,370	3.77 Mb	Deletion	Unknown	Heterozygous	Pathogenic
369755	4:980,635–2,537,887	1.56 Mb	Duplication	De novo	Heterozygous	Pathogenic / Full
376193	4:891,180–10,629,348	9.74 Mb	Deletion	De novo	Heterozygous	Pathogenic / Full
391174	4:10,001–11,322,107	11.31 Mb	Deletion	Imbalance from balanced rearrangement	Heterozygous	Likely pathogenic
392071	4:10,001–11,322,107	11.31 Mb	Duplication	Imbalance from balanced rearrangement	Heterozygous	Likely pathogenic
392074	4:10,001–11,322,107	11.31 Mb	Deletion	Imbalance from balanced rearrangement	Heterozygous	Likely pathogenic
392252	4:10,001–11,322,107	11.31 Mb	Deletion	Imbalance from balanced rearrangement	Heterozygous	Likely pathogenic
392316	4:10,001–11,322,107	11.31 Mb	Deletion	Imbalance from balanced rearrangement	Heterozygous	Likely pathogenic
392649	4:10,001–11,322,107	11.31 Mb	Deletion	Imbalance from balanced rearrangement	Heterozygous	Likely pathogenic
394818	4:10,001–11,322,107	11.31 Mb	Duplication	Imbalance from balanced rearrangement	Heterozygous	Likely pathogenic
395150	4:10,001–4,380,201	4.37 Mb	Deletion	De novo	Heterozygous	Likely pathogenic
395315	4:10,001–4,380,201	4.37 Mb	Deletion	De novo	Heterozygous	Likely pathogenic
395518	4:10,001–11,322,107	11.31 Mb	Deletion	De novo	Heterozygous	Likely pathogenic

395519	4:10,001–11,322,107	11.31 Mb	Deletion	De novo	Heterozygous	Likely pathogenic
395522	4:10,001–15,822,108	15.81 Mb	Deletion	De novo	Heterozygous	Likely pathogenic
395544	4:10,001–11,322,107	11.31 Mb	Deletion	De novo	Heterozygous	Likely pathogenic
395584	4:10,001–11,322,107	11.31 Mb	Deletion	De novo	Heterozygous	Likely pathogenic
395676	4:10,001–4,380,201	4.37 Mb	Deletion	Unknown	Heterozygous	Likely pathogenic
395932	4:10,001–4,380,201	4.37 Mb	Deletion	De novo	Heterozygous	Likely pathogenic
396084	4:10,001–4,380,201	4.37 Mb	Deletion	Imbalance from balanced rearrangement	Heterozygous	Likely pathogenic
396126	4:10,001–4,380,201	4.37 Mb	Deletion	Unknown	Heterozygous	Likely pathogenic
396158	4:10,001–19,022,108	19.01 Mb	Deletion	De novo	Heterozygous	Likely pathogenic
396162	4:10,001–4,380,201	4.37 Mb	Deletion	De novo	Heterozygous	Likely pathogenic
396174	4:10,001–4,380,201	4.37 Mb	Deletion	Unknown	Heterozygous	Likely pathogenic
396178	4:10,001–4,380,201	4.37 Mb	Deletion	Unknown	Heterozygous	Likely pathogenic
396193	4:10,001–15,822,108	15.81 Mb	Deletion	De novo	Heterozygous	Likely pathogenic
396208	4:10,001–4,380,201	4.37 Mb	Deletion	Imbalance from balanced rearrangement	Heterozygous	Likely pathogenic
396335	4:10,001–4,380,201	4.37 Mb	Deletion	De novo	Heterozygous	Likely pathogenic
396344	4:10,001–11,322,107	11.31 Mb	Deletion	De novo (mosaic)	Heterozygous	Likely pathogenic
396544	4:10,001–4,380,201	4.37 Mb	Deletion	De novo	Heterozygous	Likely pathogenic
397153	4:10,001–5,780,201	5.77 Mb	Deletion	Imbalance from balanced rearrangement	Heterozygous	Likely pathogenic
397187	4:10,001–4,380,201	4.37 Mb	Deletion	Maternal	Heterozygous	Likely pathogenic
397193	4:10,001–5,780,201	5.77 Mb	Deletion	Imbalance from balanced rearrangement	Heterozygous	Likely pathogenic
397201	4:10,001–5,780,201	5.77 Mb	Deletion	Imbalance from balanced rearrangement	Heterozygous	Likely pathogenic
397238	4:10,001–5,780,201	5.77 Mb	Deletion	Imbalance from balanced rearrangement	Heterozygous	Likely pathogenic
397400	4:10,001–4,380,201	4.37 Mb	Deletion	Unknown	Heterozygous	Likely pathogenic
397400	4:10,001–11,322,107	11.31 Mb	Duplication	Unknown	Heterozygous	Likely pathogenic
398024	4:10,001–4,380,201	4.37 Mb	Deletion	De novo	Heterozygous	Likely pathogenic
398055	4:10,001–11,322,107	11.31 Mb	Deletion	De novo	Heterozygous	Likely pathogenic
398064	4:10,001–4,380,201	4.37 Mb	Deletion	Imbalance from balanced rearrangement	Heterozygous	Likely pathogenic
398341	4:10,001–19,022,108	19.01 Mb	Deletion	De novo	Heterozygous	Likely pathogenic
398357	4:10,001–4,380,201	4.37 Mb	Deletion	Unknown	Heterozygous	Likely pathogenic
398379	4:10,001–4,380,201	4.37 Mb	Deletion	Unknown	Heterozygous	Likely pathogenic
398412	4:10,001–4,380,201	4.37 Mb	Deletion	Unknown	Heterozygous	Likely pathogenic
398421	4:10,001–4,380,201	4.37 Mb	Deletion	Unknown	Heterozygous	Likely pathogenic
399088	4:10,001–4,380,201	4.37 Mb	Deletion	Imbalance from balanced rearrangement	Heterozygous	Likely pathogenic
421082	4:64,685–24,062,099	24.00 Mb	Duplication	Unknown	Heterozygous	Likely pathogenic
472306	4:957,391–8,188,492	7.23 Mb	Deletion	Unknown	Heterozygous	Pathogenic

472457	4:49,556–2,558,263	2.51 Mb	Deletion	Unknown	Heterozygous	Pathogenic
472852	4:643,724–10,483,953	9.84 Mb	Deletion	Unknown	Heterozygous	Pathogenic
484757	4:45,886–8,886,854	8.84 Mb	Duplication	Unknown	Heterozygous	Likely pathogenic / Partial
500840	4:72,428–3,421,010	3.35 Mb	Deletion	Unknown	Heterozygous	Pathogenic
501134	4:72,428–5,883,654	5.81 Mb	Deletion	De novo (confirmed)	Heterozygous	Pathogenic
530484	4:1–4,170,606	4.17 Mb	Deletion	Unknown	Heterozygous	Pathogenic
534993	4:72,447–8,732,731	8.66 Mb	Deletion	Unknown	Heterozygous	Pathogenic / Full
564568	4:49,556–9,908,376	9.86 Mb	Deletion	De novo (confirmed)	Heterozygous	Pathogenic
564575	4:49,556–10,124,184	10.07 Mb	Deletion	De novo (confirmed)	Heterozygous	Pathogenic
564579	4:49,556–3,340,410	3.29 Mb	Deletion	De novo (confirmed)	Heterozygous	Pathogenic
564582	4:49,556–4,165,406	4.12 Mb	Deletion	De novo (confirmed)	Heterozygous	Pathogenic
564584	4:49,556–9,854,891	9.81 Mb	Deletion	De novo (confirmed)	Heterozygous	Pathogenic
564585	4:49,556–1,993,258	1.94 Mb	Deletion	De novo (confirmed)	Heterozygous	Pathogenic
564589	4:49,556–3,191,574	3.14 Mb	Deletion	Imbalance from balanced rearrangement	Heterozygous	Pathogenic
564590	4:933,992–12,593,514	11.66 Mb	Duplication	De novo (confirmed)	Heterozygous	Likely pathogenic
564593	4:823,356–6,920,690	6.10 Mb	Deletion	De novo (confirmed)	Heterozygous	Pathogenic
564594	4:49,556–18,998,677	18.95 Mb	Deletion	De novo (confirmed)	Heterozygous	Pathogenic
564595	4:1,172,836–1,965,240	792 kb	Deletion	De novo (confirmed)	Heterozygous	Pathogenic
564597	4:49,556–9,421,524	9.37 Mb	Deletion	De novo (confirmed)	Heterozygous	Pathogenic
564600	4:49,556–9,403,086	9.35 Mb	Deletion	De novo (confirmed)	Heterozygous	Uncertain significance
564602	4:49,556–3,535,488	3.49 Mb	Deletion	De novo (confirmed)	Heterozygous	Pathogenic
564613	4:49,556–10,978,557	10.93 Mb	Deletion	De novo (confirmed)	Heterozygous	Pathogenic
564617	4:49,556–4,900,232	4.85 Mb	Deletion	De novo (confirmed)	Heterozygous	Pathogenic
564620	4:142,049–22,819,023	22.68 Mb	Deletion	De novo (confirmed)	Heterozygous	Pathogenic
564621	4:70,489–4,814,577	4.74 Mb	Deletion	De novo (confirmed)	Heterozygous	Pathogenic
564623	4:49,556–21,290,590	21.24 Mb	Deletion	De novo (confirmed)	Heterozygous	Pathogenic
564624	4:49,556–9,887,824	9.84 Mb	Deletion	De novo (confirmed)	Heterozygous	Pathogenic
564628	4:71,411–5,637,072	5.57 Mb	Deletion	De novo (confirmed)	Heterozygous	Pathogenic
564630	4:70,489–9,887,824	9.82 Mb	Deletion	Imbalance from balanced rearrangement	Heterozygous	Pathogenic
564631	4:70,489–9,445,117	9.37 Mb	Deletion	Imbalance from balanced rearrangement	Heterozygous	Pathogenic
564632	4:49,556–7,885,773	7.84 Mb	Deletion	De novo (confirmed)	Heterozygous	Pathogenic
564633	4:49,556–15,691,331	15.64 Mb	Deletion	De novo (confirmed)	Heterozygous	Pathogenic
564642	4:49,556–8,918,488	8.87 Mb	Deletion	De novo (confirmed)	Heterozygous	Pathogenic
564644	4:49,556–8,355,918	8.31 Mb	Deletion	De novo (confirmed)	Heterozygous	Pathogenic

564649	4:49,556–6,094,360	6.04 Mb	Deletion	De novo (confirmed)	Heterozygous	Pathogenic
564655	4:760,156–2,515,720	1.76 Mb	Deletion	De novo (confirmed)	Heterozygous	Pathogenic
564659	4:416,226–15,813,191	15.40 Mb	Deletion	De novo (confirmed)	Heterozygous	Pathogenic
564666	4:49,556–13,899,410	13.85 Mb	Deletion	De novo (confirmed)	Heterozygous	Pathogenic
564668	4:49,556–1,935,170	1.89 Mb	Deletion	De novo (confirmed)	Heterozygous	Pathogenic
564675	4:68,453–3,405,967	3.34 Mb	Deletion	De novo (confirmed)	Heterozygous	Pathogenic
564679	4:49,556–5,017,998	4.97 Mb	Deletion	De novo (confirmed)	Heterozygous	Pathogenic
564681	4:49,556–12,006,787	11.96 Mb	Deletion	De novo (confirmed)	Heterozygous	Pathogenic
564682	4:49,556–8,867,639	8.82 Mb	Deletion	Imbalance from balanced rearrangement	Heterozygous	Pathogenic
564705	4:49,556–11,748,376	11.70 Mb	Deletion	De novo (confirmed)	Heterozygous	Pathogenic
564706	4:49,556–4,163,608	4.11 Mb	Deletion	De novo (confirmed)	Heterozygous	Pathogenic
569457	4:124,671–2,378,027	2.25 Mb	Duplication	Imbalance from balanced rearrangement	Heterozygous	–

References for supplementary tables

1. Beck DB, Cho MT, Millan F, Yates C, Hannibal M, O'Connor B, et al. A recurrent de novo CTBP1 mutation is associated with developmental delay, hypotonia, ataxia, and tooth enamel defects. Neurogenetics. 2016;17(3):173–8.
2. Beck DB, Subramanian T, Vijayalingam S, Ezekiel UR, Donkervoort S, Yang ML, et al. A pathogenic CtBP1 missense mutation causes altered cofactor binding and transcriptional activity. neurogenetics. 2019;20(3):129–43.
3. Sommerville EW, Alston CL, Pyle A, He L, Falkous G, Naismith K, et al. De novo CTBP1 variant is associated with decreased mitochondrial respiratory chain activities. Neurology: Genetics. 2017;3(5):e187.
4. Wong WK, Balasubramaniam S, Wong RSH, Graf N, Thorburn DR, McFarland R, et al. Mitochondrial respiratory chain dysfunction in a patient with a heterozygous de novo CTBP1 variant. JIMD Rep. 2022;63(6):546–54.
